# Interpretable machine learning models for bladder cancer overall survival prediction development and external validation via SEER database and Chinese cohort analysis

**DOI:** 10.1007/s12672-026-05099-6

**Published:** 2026-05-20

**Authors:** Saimaitikari Abudoubari, Abudouresuli Tuersun, Sailidan Mutailipu, Wenbin Chen, Qiange Li, Mayidili Nijiati, Xiaoguang Zou

**Affiliations:** 1 Xinjiang Key Laboratory of Artificial Intelligence Assisted Imaging Diagnose, The First People’s Hospital of Kashi Prefecture, No. 120, Yingbing Road, Kashi, Xinjiang China; 2 Department of Radiology, The First People’s Hospital of Kashi Prefecture, Kashi, China; 3 Department of Radiology, Shule County People’s Hospital in Kashgar Prefecture, Kashi, China; 4 Disease Prevention and Control Center of Shache County, Kashi, Chine

**Keywords:** Bladder cancer, Interpretable machine learning, Overall survival, SurvSHAP

## Abstract

**Objective:**

We developed interpretable machine learning(ML) models to predict overall survival in bladder cancer patients. This approach aims to improve the interpretability and transparency of our modeling results.

**Methods:**

We collected clinical and pathological information on bladder cancer patients from the SEER database, allocating it to training and validation sets in a 7:3 ratio. At the same time, we obtained an external validation cohort from Kashgar First People’s Hospital in Xinjiang, China. We performed LASSO regression and Cox regression analyses to identify relevant risk factors and then combined these to develop CoxPH and six ML models: Random Survival Forest(RSF), Gradient Boosting with Component Linear(GLMboost), decision tree(dt), boosted tree(bt), DeepSurv, and neural multi-task logistic regression(NMTLR). We evaluated the predictive performance of these ML models using the consistency index (C-index), the area under the cumulative/dynamic curve (AUC) and the integrated Brier score and Kolmogorov-Smirnov(KS). For interpretability assessment, we employed three complementary methods: (1)time-dependent variable importance to quantify feature contribution across follow-up periods; (2)partial correlation survival plots to visualize individual variable effects; and (3)aggregated survival SHapley additive interpretation(SurvSHAP) plots with mean absolute deviation metrics to validate feature impact stability at both individual and population levels.

**Results:**

The final ML model consists of 14 factors: the patient’s age, AJCCStage, chemotherapy, Mstage, marital, Tstage, bone metastasis(BoneMets), stage, radiation, histology, liverMets, Nstage, sex. Our predictive models demonstrates significant discriminative ability, with the boosting tree model performing the best. The AUC for 1-year, 3-year, and 5-year overall survival (OS) was above 0.770 for the training set, validation set, and external validation set, respectively, with the overall Brier score consistently below 0.180. The interpretability analysis of the boosting trees model further indicated that AJCCStage, age, chemotherapy, stage, Mstage, marital were the most influential predictors via quantifiable SurvSHAP values and time-dependent importance weights, with their effects visually validated through partial correlation survival curves.

**Conclusions:**

The boosting trees model prognostic model has the best performance and can be used to predict OS in bladder cancer patients, helping physicians to accurately assess patients’ overall survival rates, and providing valuable and important references for patient diagnosis, treatment, and prognosis evaluation.

**Supplementary Information:**

The online version contains supplementary material available at 10.1007/s12672-026-05099-6.

## Introduction

Bladder cancer is a common malignant tumor of the urinary system, with its global incidence posing significant concern. According to the latest statistical data, approximately 550,000 new cases of bladder cancer are diagnosed annually [1]. Incidence rates vary considerably across geographic regions: Europe and North America have the highest rates, which are three times higher than those in Southeast Asia (excluding Japan), Latin America, and North Africa [2]; while sub-Saharan Africa, Mexico, and some Middle Eastern and Central Asian countries have the lowest rates [1]. Research indicates that the lifetime risk of developing bladder cancer is 1.1% for men and 0.27% for women [3]. Mortality rates for bladder cancer show relatively little variation globally, with studies showing approximately 200,000 patients died from the disease worldwide in 2018 [3]. In 2020, the United States recorded 81,400 new bladder cancer cases and 17,980 bladder cancer deaths [4]. By 2023, the U.S. is projected to see 82,290 new bladder cancer cases and 16,710 deaths. Male bladder cancer ranks fourth among all malignancies in incidence and eighth in mortality [5]. Despite current advances in bladder cancer diagnosis and treatment—including precise cystoscopic biopsy, radical cystectomy, and novel immune checkpoint inhibitor therapy—clinical practice still lacks efficient tools to accurately distinguish high-risk from low-risk patients. This leads to over-treatment or under-treatment for some patients. Accurate prediction of overall survival (OS) is the core prerequisite for optimizing treatment decisions, reducing healthcare resource waste, and improving patient outcomes [6].

With the accumulation of medical big data and the iterative development of artificial intelligence technologies, the application advantages of machine learning in tumor prognosis prediction have become increasingly prominent. Compared to traditional statistical methods such as the Cox proportional hazards model, machine learning models can more efficiently handle nonlinear associations, interaction effects, and high-dimensional features in clinical data, demonstrating superior predictive performance in fields like lung cancer and breast cancer [7]. However, existing research on bladder cancer prognosis prediction still faces significant limitations: On one hand, some studies rely solely on a single dataset for model construction, lacking external validation and resulting in insufficient generalization capabilities; On the other hand, most machine learning models (e.g., deep neural networks, ensemble tree models) remain “black box” models. While they can output survival probability predictions, they cannot clearly explain the contribution of each clinical variable to the prediction results, nor can they identify the key risk factors affecting patient prognosis [8]. This lack of interpretability severely limits the models’ clinical translation value—clinicians cannot trust model outputs nor integrate them into personalized treatment planning if they cannot trace the reasoning behind predictive outcomes [9].

The rise of explainable machine learning offers new approaches to overcoming this challenge. By integrating modules such as feature importance assessment, local interpretability maps, and specialized survival analysis tools (e.g., SurvSHAP, Partial Survival Plots), these models preserve the high predictive accuracy of machine learning while translating complex predictive mechanisms into intuitive, clinically understandable information, thereby making the “black box” transparent [10]. Currently, explainable machine learning has been successfully applied in prognostic prediction for gastrointestinal tumors like colorectal and gastric cancers, aiding clinicians in identifying high-risk populations and optimizing follow-up strategies. However, dedicated research for bladder cancer remains scarce—particularly lacking explainable prognostic models based on multicenter data that encompass both Eastern and Western populations. This gap fails to meet the demand for precise, transparent predictive tools in clinical practice across different regions [11].

Given the current landscape, this study aims to construct and validate an interpretable machine learning survival prediction model for bladder cancer patients, focusing on addressing two core issues in existing research: “poor generalization ability” and “low interpretability”. The study will first extract large-scale clinical and pathological data of bladder cancer patients from the US Surveillance, Epidemiology, and End Results (SEER) database. Concurrently, a bladder cancer patient cohort from the First People’s Hospital of Kashgar, Xinjiang, China, will be incorporated as an external validation set to ensure the model’s applicability across diverse populations. Subsequently, CoxPH and six ML models will be constructed based on key clinical variables. The optimal model will be selected using metrics such as time-dependent Area Under the Curve (AUC) and Brier score. Finally, time-dependent feature importance analysis, partial survival plots, and the SurvSHAP global interpretation tool will be employed to systematically decipher the model’s predictive logic and identify critical prognostic factors. This study aims to provide reliable tools for risk stratification, personalized treatment planning, and prognosis assessment in bladder cancer patients, while establishing a reference paradigm for the application of interpretable machine learning in urological oncology.

## Materials and methods

### Data sources

Clinical data pertaining to patients diagnosed with bladder cancer during the period from 2013 to 2020 were retrospectively analysed in the present study. Eligible patient records were retrieved from the Surveillance, Epidemiology, and End Results (SEER) database by a two-step screening strategy based on the International Classification of Diseases for Oncology, Third Edition (ICD-O-3) system: (1) site code C67.0-C67.9 (corresponding to the anatomical site of the bladder, including the bladder neck, trigone, lateral wall, posterior wall, dome, ureteric orifice, and overlapping lesions of the bladder) and (2) histology codes 8010–8049, 8050–8089, 8120–8139, 8140–8389 (covering the main malignant epithelial neoplasms of the bladder included in this study: 8010–8049 for epithelial neoplasms not otherwise specified, 8050–8089 for squamous cell neoplasms, 8120–8139 for transitional cell papillomas and carcinomas, and 8140–8389 for adenomas and adenocarcinomas). This coding selection strictly follows the SEER database’s standard operating procedures for bladder cancer cohort extraction and the diagnostic criteria for urinary system malignant tumors in the ICD-O-3 classification system, and is consistent with the actual pathological subtype screening criteria of this study. SEER*Stat software (Version 8.4.1.2), a specialized tool for accessing SEER database resources, is freely available for download via the official SEER website (https://seer.cancer.gov/seerstat/download/). To guarantee the reliability and integrity of the study dataset, a set of exclusion criteria was strictly applied, encompassing four key conditions: (1) unavailable information on overall survival months; (2) Aged < 15 years at diagnosis; (3) missing or incomplete tumour staging data in accordance with the American Joint Committee on Cancer (AJCC) TNM classification system; (4) undetermined histological grade of the tumour; and (5) incomplete records of SEER comprehensive summary stage.

**Fig. 1:**
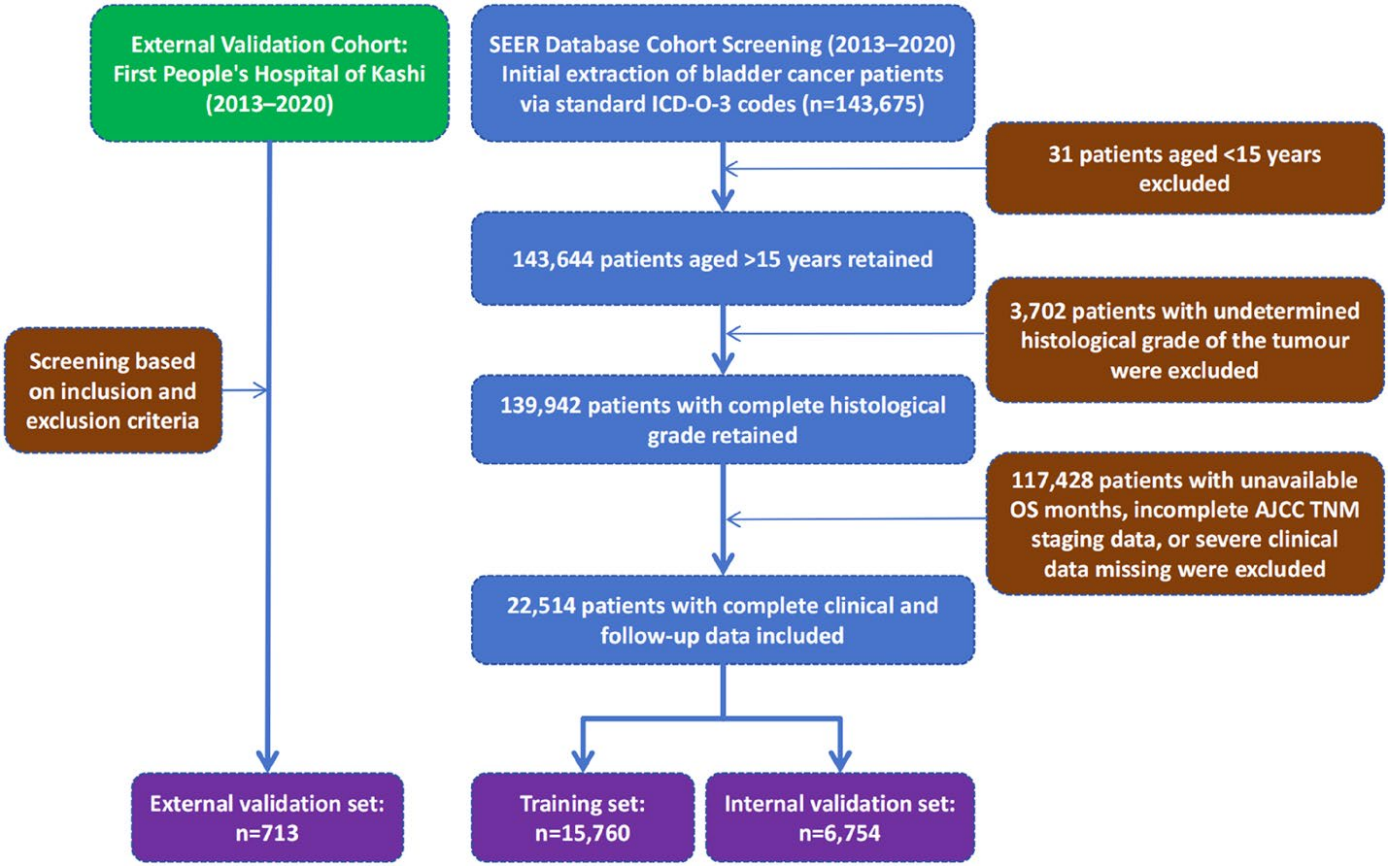
Study cohort screening flow chart of bladder cancer patients (2013–2020)

Based on the predefined inclusion and exclusion criteria, a total of 22,514 bladder cancer patients were ultimately enrolled in this study. The enrolled cohort was randomly stratified into a training set and an internal validation set at a 7:3 ratio, with 15,760 cases allocated to the training set and 6,754 cases to the internal validation set. Furthermore, an external validation cohort consisting of 713 patients was recruited from Kashgar First People’s Hospital, with all patients receiving treatment for bladder cancer at this institution between 2013 and 2020. Data collection throughout the study was performed by three independent researchers, following a dual-extraction and cross-verification protocol: two researchers were responsible for separate data extraction, and the third conducted rigorous verification to ensure the accuracy and consistency of the collected data. Notably, all clinical data of the included patients were fully anonymized to protect patient privacy, and the requirement for informed consent was waived for this retrospective study (Fig. 1).

### Variable set

Given the potential for incomplete clinical data entries in the SEER database, a standardized and rigorous missing data handling strategy was implemented prior to cohort stratification to ensure the integrity and reliability of the study dataset. First, a comprehensive missing rate assessment was conducted for all initially collected clinical variables of bladder cancer patients. Variables with a missing rate exceeding 5% were excluded to avoid potential bias caused by a high proportion of missing values, and a total of 17 clinical variables with a missing rate of less than 5% were finally retained for subsequent analysis, including age at diagnosis (years), sex, marital status (married, single, separated, widowed), primary Site(Bladder; Lateral wall of bladder; Overlapping lesion of bladder; Posterior wall of bladder; Trigone of bladder; Dome of bladder; Bladder neck; Ureteric orifice), Histology[transitional cell papillomas and carcinomas(TCP); squamous cell neoplasms(SCN); epithelial neoplasms(EN); adenomas and adenocarcinomas(ADC)], grade (G1; G2; G3; G4), SEER stage (localized, regional, distant), AJCCstage, AJCC 7th edition TNM staging, metastasis(bone, liver, lung), and treatment modality (surgery, radiotherapy, and chemotherapy). The primary outcome measure in this study was overall survival (OS), defined as the time interval between the date of diagnosis and the occurrence of death or the most recent follow-up.

For the small amount of missing values in the retained 17 variables, targeted imputation methods were adopted according to the data type: mode imputation was used to fill in missing values for categorical variables (e.g., marital status, treatment modality, metastasis status, histological subtype), which could maintain the original distribution characteristics of discrete clinical indicators; mean imputation was applied for the only continuous variable (age at diagnosis), based on the normal distribution characteristics of age in the bladder cancer cohort.

All missing data assessment, variable screening and imputation operations were independently completed by two researchers using Python 3.12.9 (Pandas and Scikit-learn toolkits), and a third researcher conducted cross-verification to ensure the consistency and accuracy of the results. The above missing data handling strategy was uniformly applied to the training set, internal validation set and external validation set to eliminate selection bias caused by inconsistent preprocessing methods.

### Determining prognostic factors for survival

All the variables in this study were converted into categorical variables and expressed as frequencies and proportions. To mitigate overfitting, the most relevant predictive features were selected using the least absolute shrinkage and selection operator (LASSO) method. Cox proportional hazards models were then used for multivariate analysis to determine important prognostic factors. The corresponding 95% confidence intervals (CIs) were calculated for all potential risk factors.

### Model development

In the present study, a traditional Cox proportional hazards regression model and six distinct machine learning algorithms—Random Survival Forest (RSF), Gradient Boosting with Component Linear Models (GLMboost), decision tree, boosted tree, DeepSurv, and Neural Multi-Task Logistic Regression (NMTLR)—were constructed and validated to establish prognostic models for bladder cancer patients.

RSF integrates the framework of random forests with survival analysis methodologies, making it well-suited for processing right-censored survival data. This algorithm innovates by adopting novel survival splitting criteria for the generation of survival trees and developing tailored algorithms to impute missing data points. Additionally, RSF adheres to the event retention principle inherent to survival forest models, which is utilised to calculate the overall mortality rate—a straightforward and interpretable metric that serves as a key predictor in prognostic analysis. All computations for the RSF model were implemented via the random forest SRC (rfsrc) package in R software.

GLMboost is a regression and classification algorithm built on the gradient boosting tree framework, with the general linear model as its foundational base learner. Adopting an incremental gradient boosting strategy, GLMboost enhances the predictive performance of the base model in a stepwise manner, while simultaneously mitigating model complexity through regularisation techniques. A notable advantage of GLMboost is its strong adaptability to diverse data types, including both categorical and continuous variables. Moreover, this algorithm exhibits robust capacity to address missing data issues, a common challenge in the analysis of real-world clinical datasets.

Decision trees (DT) generate simple, interpretable decision rules from raw clinical data to predict the values of dependent variables in survival analysis. In contrast, boosted trees represent an ensemble learning approach that fuses predictive outputs from multiple base models to elevate the overall performance and generalisation ability of the prognostic model. While decision trees partition the dataset by selecting the most discriminative features at each node, boosted tree ensembles integrate predictions from multiple individually trained decision trees to achieve superior predictive performance on unseen data.

As an ensemble learning method, boosted trees (BT) iteratively generate a series of weak learners and aggregate their outputs to form a high-performance strong learner, thereby improving the accuracy of survival prediction. Unlike decision trees, which are constructed independently without considering prior model errors, boosted trees are trained in an iterative fashion: each training round prioritises the samples that were misclassified or poorly predicted in the previous iteration, with the aim of gradually reducing model bias and enhancing predictive accuracy. In the context of survival analysis, boosted trees optimise model parameters by minimising a predefined loss function (e.g., the negative log-likelihood function), rendering them particularly applicable to modelling nonlinear relationships and analysing high-dimensional clinical data.

DeepSurv is a deep learning-based survival analysis model that integrates the traditional Cox proportional hazards model with deep neural networks, enabling the automatic identification of complex nonlinear interactions among high-dimensional clinical features. Compared with conventional survival analysis methods, DeepSurv demonstrates prominent advantages in processing high-dimensional sparse data and capturing intricate, non-linear feature correlations in clinical datasets. The model estimates its core parameters by maximising the partial log-likelihood function; the hidden layers of the deep neural network extract and learn more comprehensive and hierarchical feature representations from raw clinical data than traditional statistical methods.

Neural Multi-Task Logistic Regression (NMTLR) is a neural network-based model specifically designed for survival analysis, which is capable of performing survival prediction tasks across multiple time points simultaneously. By sharing underlying feature representations across different time points and learning the inherent correlations between prognostic predictions at various follow-up time points, NMTLR effectively improves the accuracy of time-dependent survival prediction. This model is built on a multi-task learning framework, where each individual task corresponds to a specific follow-up time point; for each time point, logistic regression is employed to predict the probability of the occurrence of the primary endpoint event (e.g., cancer-specific death) before that time point.

### Hyperparameter tuning

To ensure the optimal performance and reproducibility of the six machine learning models, a standardized hyperparameter tuning process was implemented for each algorithm. All tuning operations were conducted on the training set using 5-fold cross-validation (5-CV) to avoid overfitting and data leakage. Grid Search Cross-Validation (GridSearchCV) or Random Search Cross-Validation (RandomizedSearchCV) was selected based on the complexity of each model, with optimization metrics prioritizing clinical relevance and statistical robustness (C-index, integrated Brier score, and time-dependent AUC).

Random Survival Forest (RSF): Tuning focused on balancing model complexity and generalization, with final parameters set as n_estimators = 200, max_depth = 5, min_samples_split = 6, min_samples_leaf = 3, max_features=’sqrt’ via GridSearchCV (optimization metric: C-index + integrated Brier score).

Gradient Boosting with Component Linear Models (GLMboost): Adopted RandomizedSearchCV (50 iterations) to optimize n_estimators = 200 and learning_rate = 0.1, subsample = 1.0, ensuring stable performance without overfitting (optimization metric: integrated Brier score).

Decision Tree: As a baseline interpretable model, tuning was performed via GridSearchCV to balance interpretability and predictive accuracy. Key hyperparameters were optimized to max_depth = 5, min_samples_split = 10, min_samples_leaf = 5 (optimization metric: C-index), avoiding overfitting while maintaining clear decision rules.

Boosted Tree: Key hyperparameters including n_estimators = 200, learning_rate = 0.1, max_depth = 3, subsample = 0.5, min_samples_split = 2, min_samples_leaf = 1 were optimized via GridSearchCV, prioritizing time-dependent AUC (1/3/5-year) to enhance long-term survival prediction reliability.

DeepSurv: Utilized a two-layer MLP network (num_nodes=[32, 32]) with learning_rate = 0.01, batch_size = 256, dropout = 0.1, and early stopping (epochs = 100) to prevent overtraining (optimization metric: partial log-likelihood + validation C-index).

Neural Multi-Task Logistic Regression(NMTLR): Adopted num_nodes=[32, 32], num_durations = 100, learning_rate = 0.01, batch_size = 256, dropout = 0.1, optimized via early stopping and cross-validation to improve multi-time-point prediction consistency (optimization metric: validation loss + integrated Brier score).

All tuning processes were implemented using Python 3.12.9 with Scikit-learn, Scikit-survival, PyTorch, and Pycox toolkits, ensuring full reproducibility of the model construction workflow.

### Model testing and evaluation

The performance of seven different models was evaluated using various metrics, including the C-index, the area under the receiver operating characteristic curve (AUC) and the Brier score. The C-index measures the ability of survival models to discriminate, with values above 0.7 indicating practicality. A higher C-index is associated with a greater likelihood of successful model prediction. The Brier score is also used to assess the predictive accuracy of models, with values below 0.25 indicating practical application. Higher predictive accuracy is associated with a lower Brier score. Furthermore, the Kolmogorov-Smirnov (KS) test(the goodness-of-fit metrics suitable for survival analysis) were additionally incorporated. The KS test quantifies the overall discrepancy between the predicted and observed survival distributions by calculating the maximum distance between the two distributions.

### Model interpretability

The rational application of artificial intelligence in medical research necessitates intuitive interpretability for black-box machine learning models, as well as validation of their clinical practical value. To address this critical issue, we adopted a comprehensive explanatory modelling strategy incorporating global interpretability approaches and high-fitting analytical methods. Global interpretability for the entire study cohort was achieved by integrating four analytical techniques: time-dependent variable importance assessment, partial dependence analysis, survival curve plotting, and aggregated Survival SHapley Additive Explanations (SurvSHAP) visualization.

Time-dependent variable importance analysis was performed to identify core features with a significant impact on survival outcomes at different follow-up time points, which further uncovered the temporal dynamic variation characteristics of feature effects on prognostic prediction.Partial correlation survival curves were constructed to visualize the functional relationship between key clinical features and survival probability, which intuitively reflected the independent prognostic impact of a single feature after adjusting for the confounding effects of other variables.SurvSHAP, a SHAP extension method specifically developed for survival analysis scenarios, was utilized to quantitatively evaluate the contribution of each feature to the model’s predictive results, and to clarify the directional correlation between feature value changes and prognostic risk predictions (i.e., risk-increasing or risk-reducing effects).

**Fig. 2:**
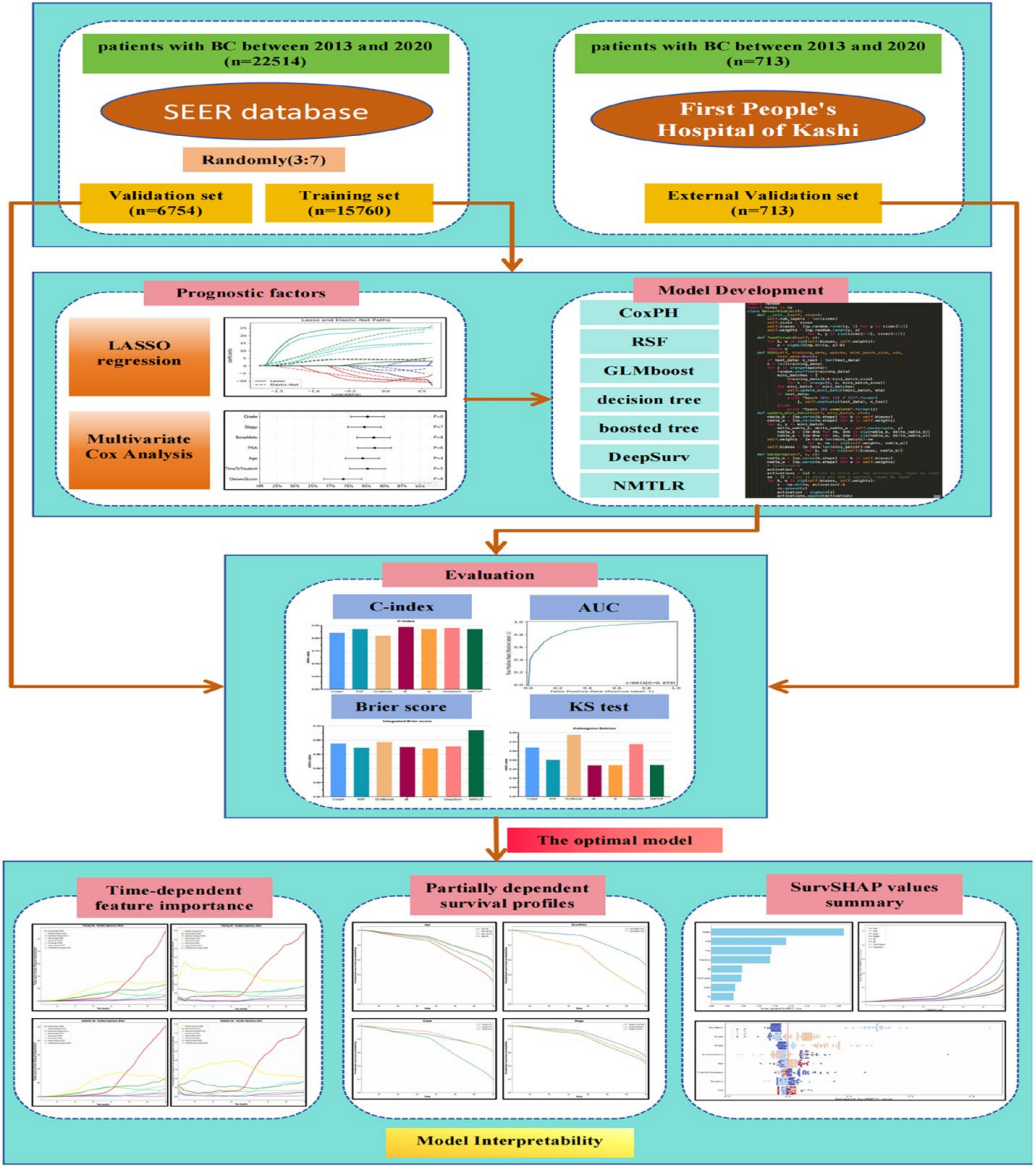
Work flow chart

The complete research framework adopted in this study is presented in Fig. 2 (Study Flow Chart).

### Statistical analysis

LASSO regression was used to screen for survival risk factors, and univariate and multivariate Cox regression analyses were performed in SPSS 26.0 to identify independent prognostic factors (statistical significance was set at *P* < 0.05). Based on the screened features, a Cox proportional hazards regression model (CoxPH) and six machine learning (ML) models were constructed, including Random Survival Forest (RSF), component-wise linear Gradient Boosting (GLMboost), Decision Tree (dt), Boosted Tree (bt), Deep Survival Network (DeepSurv), and Neural Multi-Task Logistic Regression (NMTLR). To comprehensively and accurately evaluate model performance, in addition to clinically recognized metrics—including the C-index (for assessing discriminative ability), integrated cumulative/dynamic AUC (for evaluating discriminative performance at different time points), and integrated Brier score (for measuring calibration between predicted probabilities and actual outcomes)—one goodness-of-fit metric suitable for survival analysis were additionally incorporated: the Kolmogorov-Smirnov (KS) test. For the optimal model selected, overall explanations were generated for the entire cohort using time-dependent variable importance, partial dependency analysis, survival plots and aggregated survival SHapley additive explanations (SurvSHAP) plots.

The construction of all ML models, performance evaluation (including calculation of the C-index, integrated AUC, integrated Brier score, KS test, and CvM test), and interpretability analyses were implemented using Python 3.12.9. The analysis relied on the following toolkits: data processing (Pandas, NumPy, SciPy), model construction (Scikit-learn, Lifelines, Scikit-survival, PyTorch, Torchvision), interpretability analysis (SHAP), and visualization (Matplotlib, Seaborn).

## Results

### Demographic and clinicopathological characteristics

A comprehensive cohort of 22,514 bladder cancer patients diagnosed between 2013 and 2020 was carefully selected from the SEER database using predefined inclusion and exclusion criteria. Of these patients, 15,760 were assigned to the training set and the remaining 6,754 formed the validation set. A further 713 patients treated at Kashgar First People’s Hospital from 2013 to 2020 formed an external validation set. Statistical analysis using the Pearson’s chi-square test was performed to compare the categorical clinical and pathological characteristics among the training set, internal validation set, and external validation set. The results showed that most baseline characteristics were well balanced across the three cohorts (all *P* > 0.05), with the exception of tumor grade (*P* = 0.010) and histology (*P* = 0.029), for which statistically significant differences were observed (Table 1).

**Table 1:** General clinical data of training, validation and external validation set samples of bladder cancer patients [*n*(%)]

Characteristic	Training set (*n* = 15760) [*n*(%)]	Validation set (*n* = 6754) [*n*(%)]	External validation set (*n* = 713) [*n*(%)]	Χ^2^	*P*
Sex				1.815	0.404
Male	12,174 (77.2)	5162 (76.4)	547 (76.7)		
Female	3586 (22.8)	1592 (23.6)	166 (23.3)		
Grade				16.906	0.010
G1	331 (2.1)	128 (1.9)	12 (1.7)		
G2	1327 (8.4)	612 (9.1)	75 (10.5)		
G3	2657 (16.9)	1207 (17.9)	145 (20.3)		
G4	11,445 (72.6)	4807 (71.2)	481 (67.5)		
Stage				2.452	0.653
Localized	12,150 (77.1)	5194 (76.9)	535 (75)		
Regional	2539 (16.1)	1080 (16)	127 (17.8)		
Distant	1071 (6.8)	480 (7.1)	51 (7.2)		
AJCCstage				2.212	0.899
1	7866 (49.9)	3360 (49.7)	349 (48.9)		
2	4538 (28.8)	1934 (28.6)	203 (28.5)		
3	1364 (8.7)	586 (8.7)	72 (10.1)		
4	1992 (12.6)	874 (12.9)	89 (12.5)		
Tstage				0.111	1.000
T1	8057 (51.1)	3457(51.2)	362 (50.8)		
T2	5290 (33.6)	2270(33.6)	240 (33.7)		
T3	1509 (9.6)	642(9.5)	70 (9.8)		
T4	904 (5.7)	385(5.7)	41 (5.8)		
Nstage				4.932	0.553
N0	14,312 (90.8)	6141 (90.9)	649 (91)		
N1	531 (3.4)	223 (3.3)	30 (4.2)		
N2	693 (4.4)	299 (4.4)	22 (3.1)		
N3	224 (1.4)	91 (1.3)	12 (1.7)		
Mstage				1.571	0.456
M0	14,918 (94.7)	6369 (94.3)	678 (95.1)		
M1	842 (5.3)	385 (5.7)	35 (4.9)		
Surgery				0.255	0.882
No	125 (0.8)	57 (0.8)	5 (0.7)		
Yes	15,635 (99.2)	6697 (99.2)	708 (99.3)		
Radiation				0.656	0.720
No	14,092 (89.4)	6015 (89.1)	638 (89.5)		
Yes	1668 (10.6)	739 (10.9)	75 (10.5)		
Chemotherapy				0.136	0.934
No	9423 (59.8)	4056 (60.1)	427 (59.9)		
Yes	6337 (40.2)	2698 (39.9)	286 (40.1)		
Bone Metastasis				0.832	0.660
No	15,451 (98)	6610 (97.9)	700 (98.2)		
Yes	309 (2)	144 (2.1)	13 (1.8)		
Liver Metastasis				0.023	0.988
No	15,584 (98.9)	6677 (98.9)	705 (98.9)		
Yes	176 (1.1)	77 (1.1)	8 (1.1)		
Lung Metastasis				2.089	0.352
No	15,503 (98.4)	6626 (98.1)	702 (98.5)		
Yes	257 (1.6)	128 (1.9)	11 (1.5)		
Marital				3.804	0.703
Married	9591 (60.9)	4185 (62)	427 (59.9)		
Single	1924 (12.2)	798 (11.8)	84 (11.8)		
Separated	1626 (10.3)	664 (9.8)	79 (11.1)		
Widowed	2619 (16.6)	1107 (16.4)	123 (17.3)		
Primary Site				15.019	0.377
Bladder	4869 (30.9)	2039 (30.2)	214 (30)		
Lateral wall of bladder	3664 (23.2)	1656 (24.5)	186 (26.1)		
Overlapping lesion of bladder	2301 (14.6)	969 (14.3)	104 (14.6)		
Posterior wall of bladder	1594 (10.1)	664 (9.8)	78 (10.9)		
Trigone of bladder	1227 (7.8)	507 (7.5)	50 (7)		
Dome of bladder	803 (5.1)	381 (5.6)	34 (4.8)		
Bladder neck	789 (5)	325 (4.8)	33 (4.6)		
Ureteric orifice	513 (3.3)	213 (3.2)	14 (2)		
Age(year)				6.198	0.401
< 60	2425 (15.4)	1011 (15)	124 (17.4)		
61 ~ 70	4200 (26.6)	1762 (26.1)	190 (26.6)		
71 ~ 80	4810 (30.5)	2132 (31.6)	220 (30.9)		
> 81	4325 (27.4)	1849 (27.4)	179 (25.1)		
Histology				14.042	0.029
TCP	14,860 (94.3)	6350 (94)	679 (95.2)		
SCN	400 (2.5)	182 (2.7)	12 (1.7)		
EN	275 (1.7)	144 (2.1)	18 (2.5)		
ADC	225 (1.4)	78 (1.2)	4 (0.6)		

### Analysis of prognostic factors of bladder cancer

**Fig. 3:**
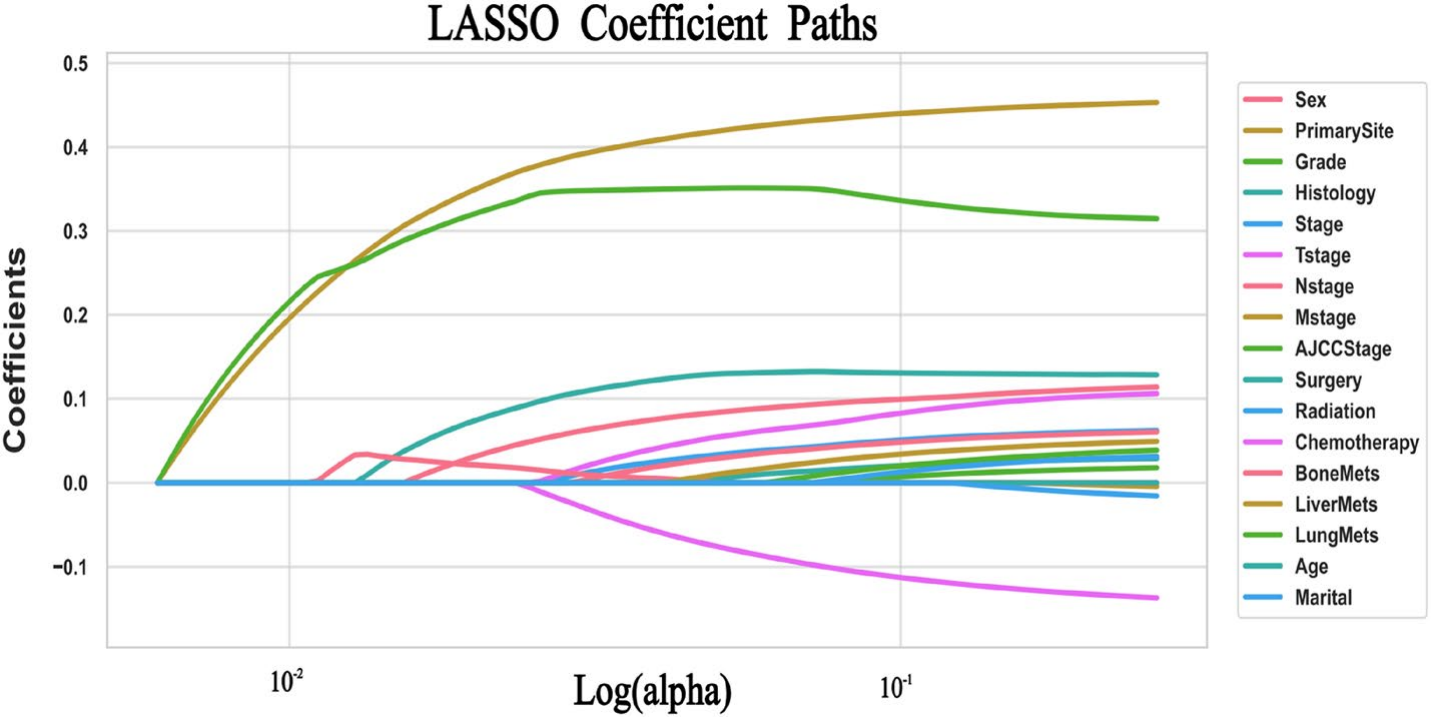
Feature selection using LASSO regression (The LASSO coefficient profiles depict the representation of 17 variables)

A total of 17 clinical parameters were included. LASSO regression analysis was used to screen parameters with *P* < 0.05, and 14 variables were retained (Table 2; Fig. 3). These 14 variables were then included in the Cox regression analysis, and the results showed that the 14 variables (age, AJCCStage, Mstage, marital, chemotherapy, Tstage, bone metastasis(BoneMets), histology, grade, radiation, Nstage, stage, liverMets, sex) had p-values below 0.05 and were identified as independent risk factors for overall survival in bladder cancer (Table 3).

**Table 2:** LASSO variable screening results table

Variable	Coefficient	Z_Score	P_Value
Age	0.462	35.684	< 0.001
AJCCStage	0.302	10.528	< 0.001
Mstage	0.130	7.053	< 0.001
Marital	0.124	11.685	< 0.001
Chemotherapy	-0.123	-10.473	< 0.001
Tstage	0.111	4.505	< 0.001
BoneMets	0.071	5.319	< 0.001
Histology	0.050	4.668	< 0.001
Grade	0.047	3.294	< 0.001
Radiation	0.040	5.580	< 0.001
Nstage	0.039	2.073	0.038
Stage	0.038	2.051	0.042
LiverMets	0.037	2.022	0.045
Sex	-0.035	-2.013	0.047
LungMets	0.026	1.530	0.126
Surgery	-0.010	-1.045	0.296
PrimarySite	< 0.001	0.336	0.737

**Table 3:** Univariate and multivariate analysis of prognostic factors related to overall survival in patients with bladder cancer

Characteristic	Univariate analysis			Multivariate analysis	
	HR (95%CI)	*P*		HR (95%CI)	*P*
Sex					
Male	Reference value			Reference value	
Female	1.156(1.103,1.211)	< 0.001		1.074(1.022,1.129)	0.005
Grade					
G1	Reference value			Reference value	
G2	1.102(0.927,1.31)	0.269		1.110(0.933,1.319)	0.239
G3	1.788(1.52,2.103)	< 0.001		1.316(1.118,1.550)	< 0.001
G4	1.641(1.402,1.92)	< 0.001		1.317(1.124,1.543)	< 0.001
Histology					
TCP	Reference value			Reference value	
SCN	1.787(1.594,2.003)	< 0.001		1.408(1.248,1.588)	< 0.001
EN	2.155(1.885,2.463)	< 0.001		1.403(1.225,1.607)	< 0.001
ADC	1.316(1.122,1.544)	< 0.001		1.134(0.965,1.333)	0.127
Stage					
Localized	Reference value			Reference value	
Regional	1.737(1.650,1.822)	< 0.001		1.034(0.908,1.177)	0.616
Distant	4.515(4.220,4.831)	< 0.001		1.337(1.076,1.663)	0.009
Tstage					
T1	Reference value			Reference value	
T2	2.027(1.939,2.119)	< 0.001		0.921(0.778,1.091)	0.343
T3	2.095(1.961,2.239)	< 0.001		0.915(0.767,1.091)	0.320
T4	3.45(3.193,3.728)	< 0.001		1.305(1.096,1.554)	0.003
Nstage					
N0	Reference value			Reference value	
N1	1.800(1.631,1.986)	< 0.001		0.872(0.740,1.028)	0.102
N2	2.668(2.457,2.899)	< 0.001		1.182(1.024,1.365)	0.022
N3	2.774(2.408,3.194)	< 0.001		1.102(0.918,1.322)	0.299
Mstage					
M0	Reference value			Reference value	
M1	4.494(4.175,4.838)	< 0.001		1.424(1.189,1.706)	< 0.001
AJCCstage					
1	Reference value			Reference value	
2	1.930(1.842,2.023)	< 0.001		2.138(1.792,2.551)	< 0.001
3	2.032(1.893,2.180)	< 0.001		2.029(1.625,2.534)	< 0.001
4	3.841(3.627,4.068)	< 0.001		3.305(2.570,4.250)	< 0.001
Radiation					
No	Reference value			Reference value	
Yes	1.930(1.823,2.044)	< 0.001		1.134(1.065,1.206)	< 0.001
Chemotherapy					
No	Reference value			Reference value	
Yes	0.890(0.854,0.927)	< 0.001		0.723(0.691,0.757)	< 0.001
Bone Metastasis					
No	Reference value			Reference value	
Yes	5.160(4.595,5.795)	< 0.001		1.598(1.380,1.852)	< 0.001
Liver Metastasis					
No	Reference value			Reference value	
Yes	6.005(5.160,6.988)	< 0.001		1.354(1.140,1.607)	< 0.001
Marital					
Married	Reference value			Reference value	
Single	1.198(1.126,1.276)	< 0.001		1.335(1.253,1.423)	< 0.001
Separated	1.162(1.086,1.243)	< 0.001		1.318(1.231,1.412)	< 0.001
Widowed	1.911(1.817,2.010)	< 0.001		1.303(1.233,1.377)	< 0.001
Age(year)					
< 60	Reference value			Reference value	
61 ~ 70	1.297(1.201,1.400)	< 0.001		1.368(1.266,1.477)	< 0.001
71 ~ 80	1.895(1.762,2.038)	< 0.001		2.044(1.897,2.201)	< 0.001
> 81	3.555(3.310,3.818)	< 0.001		3.735(3.459,4.033)	< 0.001

### Model comparison

We developed CoxPH and six ML prediction models and evaluated their performance using internal and external validation cohorts.

**Fig. 4:**
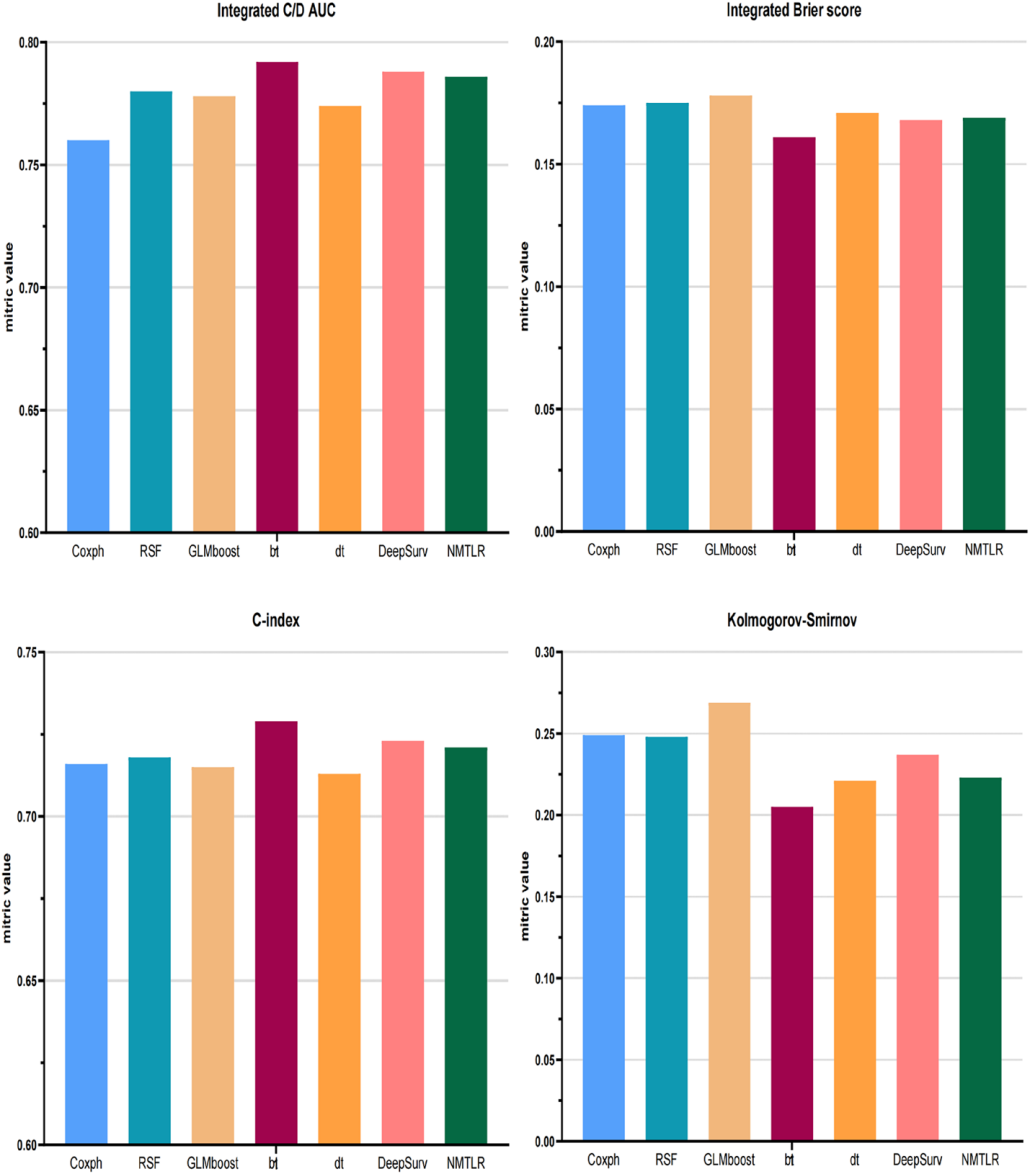
Model performance for the training set was displayed in the form of bar plots. Cox proportional hazard(Coxph); random survival forest(rfsrc); gradient boosting with component-wise linear(glmboost); boosting trees(bt); decision trees(dt); Probabilistic Survival Prediction with Deep Neural Networks(DeepSurv); neural multi-task logistic regression(NMTLR)

Model performance metrics (Table 4; Fig. 4) demonstrated that all models achieved favorable predictive performance across datasets, with integrated Brier scores below 0.19. For the training set, the bt model stood out with the integrated Brier score (0.161), highest C-index (0.729) and integrated C/D AUC (0.792); in the validation set, it maintained the integrated Brier score (0.169), the highest C-index (0.723) and integrated C/D AUC (0.779); even in the external validation set, it exhibited stable performance (integrated Brier score = 0.168, C-index = 0.718 and C/D AUC = 0.774) (Table 4; Fig. 3).

Additionally, we incorporated Kolmogorov-Smirnov (KS) metric—the statistical measure that quantify the goodness-of-fit between predicted and observed survival distributions—for more intuitive performance comparison. The bt model has the lowest KS values (training set: 0.205; validation set: 0.210; external validation set: 0.231) in all datasets, further supporting the model’s discriminative ability (Table 4; Fig. 4).

**Table 4:** The all models’ performance in the training set, validation set, and external validation set

Training set	C-index	Integrated C/D AUC	Integrated brier score	KS
Coxph	0.716	0.760	0.174	0.249
RSF	0.718	0.780	0.175	0.248
GLMboost	0.715	0.778	0.178	0.269
Boosting trees	0.729	0.792	0.161	0.205
Decision trees	0.713	0.774	0.171	0.221
DeepSurv	0.723	0.788	0.168	0.237
NMTLR	0.721	0.786	0.169	0.223
Validation set				
Coxph	0.715	0.770	0.173	0.255
RSF	0.714	0.765	0.176	0.257
GLMboost	0.713	0.769	0.178	0.277
Boosting trees	0.723	0.779	0.169	0.210
Decision trees	0.709	0.765	0.176	0.223
DeepSurv	0.719	0.771	0.170	0.238
NMTLR	0.720	0.773	0.168	0.219
External validation set				
Coxph	0.711	0.766	0.181	0.274
RSF	0.710	0.765	0.180	0.260
GLMboost	0.709	0.763	0.182	0.276
Boosting trees	0.718	0.774	0.168	0.231
Decision trees	0.708	0.767	0.176	0.236
DeepSurv	0.714	0.767	0.180	0.255
NMTLR	0.711	0.760	0.172	0.239

**Fig. 5:**
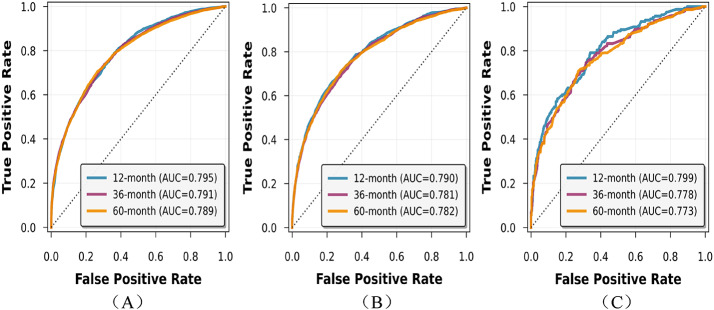
ROC curve analysis of the boosting trees model was used to evaluate the accuracy of the 1-, 3-, and 5-year predictions. **A** training set. **B** validation set. **C** external validation set

The ROC curves (Fig. 5) show that the areas under the receiver operating characteristic curve at 1 year, 3 years, and 5 years are as follows: 0.795, 0.791, and 0.789 for the training set; 0.790, 0.781, and 0.782 for the validation set; and 0.799, 0.778, and 0.773 for the external validation set.

### Model interpretibility

To further evaluate the optimal model, we performed a global analysis to gain a comprehensive understanding of its performance.

#### Time-dependent feature importance

**Fig. 6:**
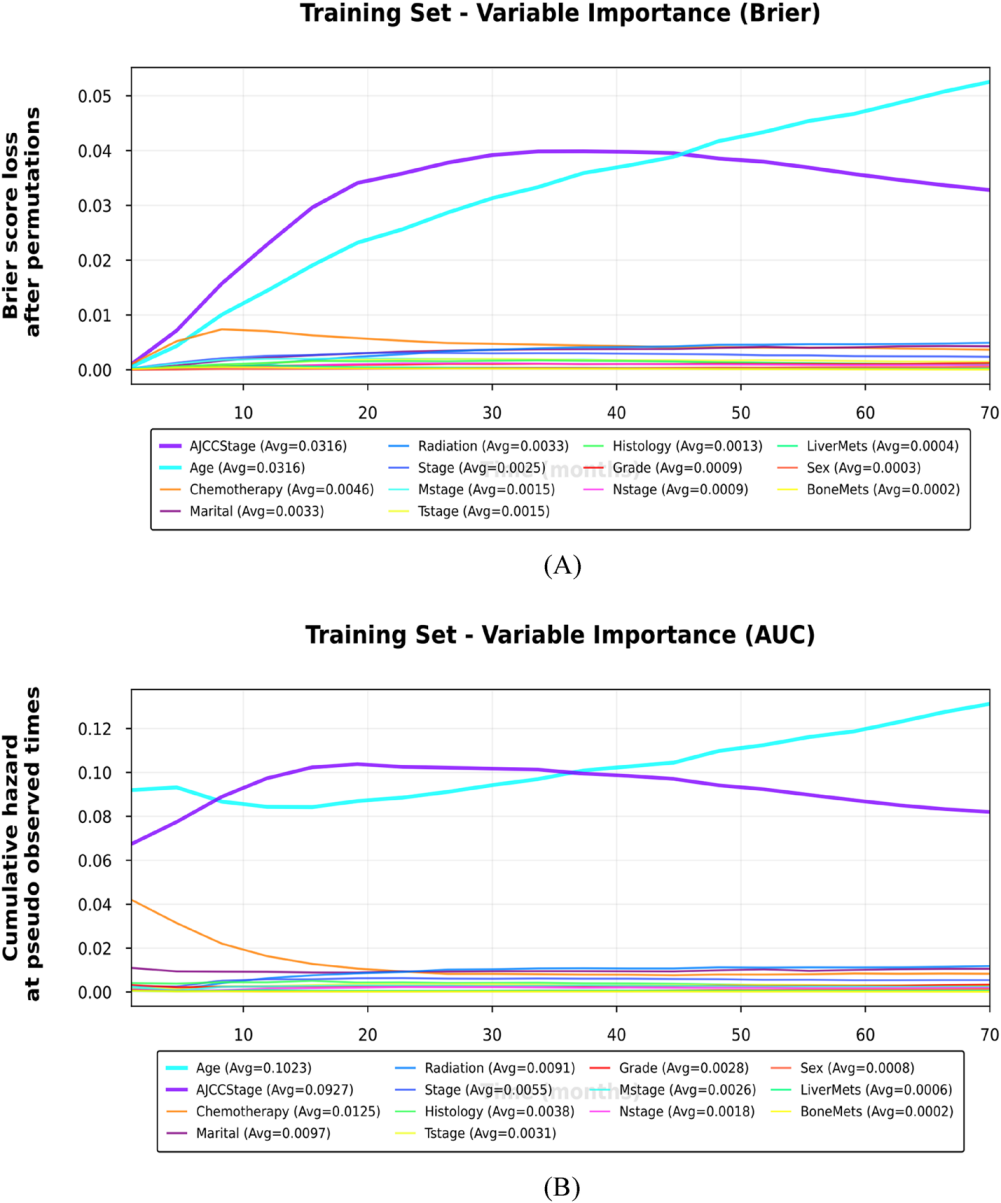
Time-dependent feature importance for the training set. **A** The Brier score loss after permutation; **B** the C/D AUC loss after permutation. The y-axis represents the variation in the loss function after permuting each covariate

In the present study, the impact of each clinical variable on the global predictive performance of the boosted tree (BT) model was systematically evaluated. Given that the prognostic influence of individual variables may vary across different follow-up time points, the time-dependent importance of each variable in the BT model was quantified using a perturbation-based approach: a specific variable was replaced with random noise (while all other variables remained unchanged), and the resulting degradation in model predictive performance was measured as an indicator of variable importance. Two key metrics were adopted to assess such performance degradation: the increment in Brier score and the reduction in time-dependent AUC following variable perturbation (detailed results are presented in Fig. 6).

Variable importance exhibited clear time-dependent patterns: a larger increase in Brier score (or larger decrease in AUC) after replacing a variable indicated that this variable had a more critical role in predicting OS at that specific time point. Specifically, the Brier score-based analysis revealed more distinct time-dependent patterns. Our results demonstrated that the American Joint Committee on Cancer (AJCC) stage exerted the most prominent impact on OS prediction when the follow-up time was less than approximately 45 months—replacing this variable led to the most significant deterioration in model performance, as reflected by the largest increase in Brier score and the most substantial decrease in time-dependent AUC. In contrast, after 45 months of follow-up, age emerged as the dominant prognostic predictor, with its replacement resulting in the most remarkable decline in model predictive ability.

These time-dependent variable importance patterns hold substantial clinical implications for the personalized management of bladder cancer patients:​.

For patients with a predicted survival time of the predominant influence of AJCC stage suggests that priority should be given to early interventions targeting the extent of disease—such as systemic therapy or multimodal treatment strategies—to improve short-term survival outcomes. Additionally, close monitoring of disease progression in this patient subgroup may facilitate the timely adjustment of treatment regimens.

For patients who survived beyond 45 months, the dominant role of age indicates that patient age remains a key prognostic driver in the mid-to-long term. This finding supports the clinical significance of age-adapted surveillance and management protocols, as elderly patients may require tailored therapeutic interventions and supportive care measures to prolong survival.​.

Collectively, these results highlight that the prognostic factors associated with bladder cancer exhibit dynamic changes over time, underscoring the necessity of time-stratified risk assessment and adaptive treatment planning in clinical practice.

#### Partial dependence survival profiles

**Fig. 7:**
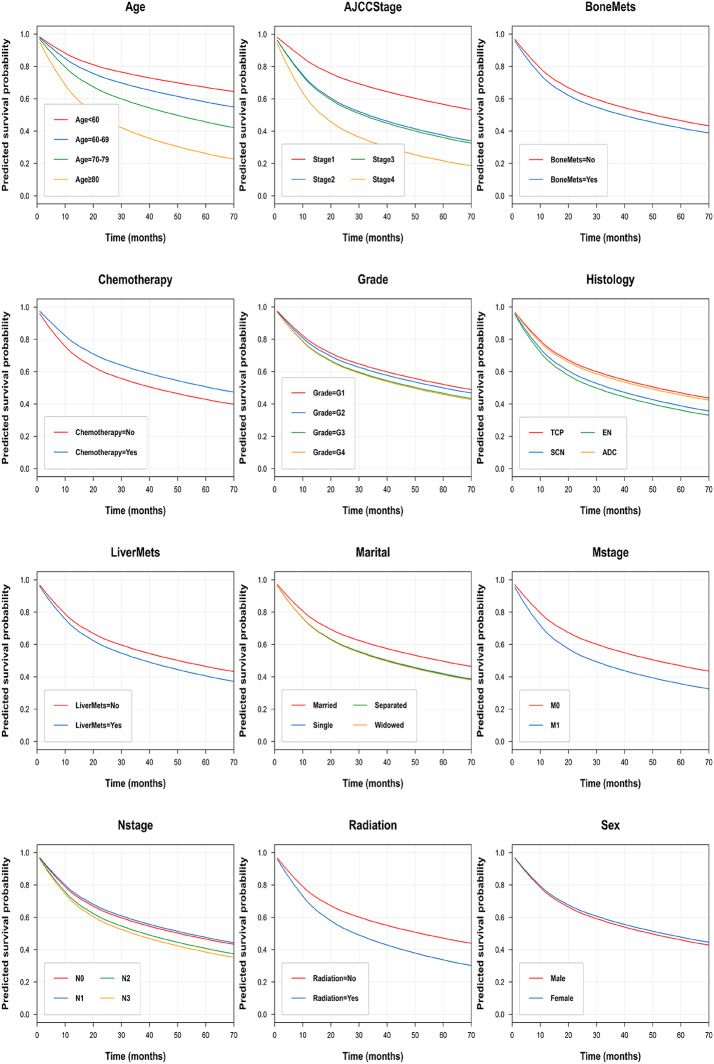

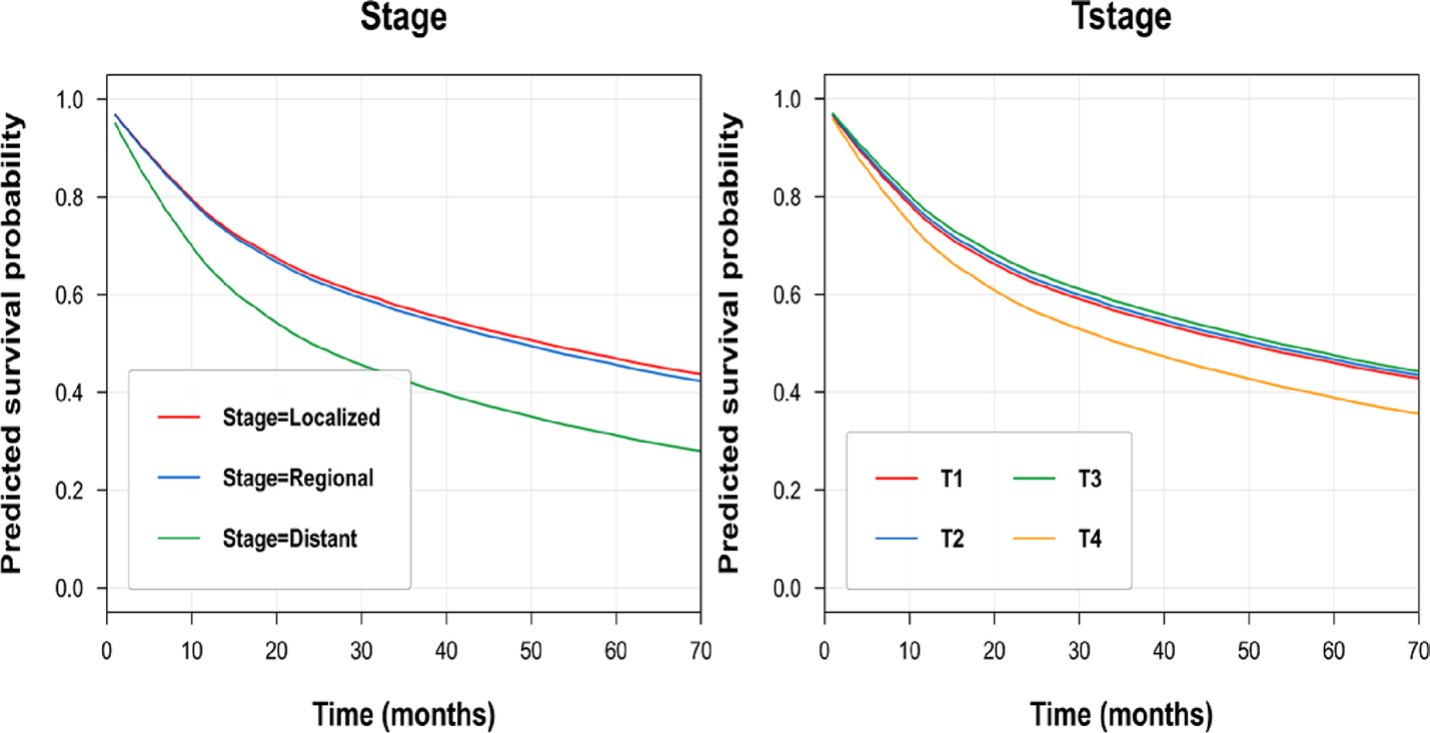
PDP can provide a global explanation for the boosting trees model. The survival function values of any covariates are depicted on the y-axis. The larger the differences between levels of a factor, the greater the impact of the same factor on OS. A lower numerical value indicates a lower probability of survival

Partial dependence survival profiles (PDPs) were further utilized to deliver a global interpretive analysis of the boosted tree (BT) model, with the corresponding results illustrated in Fig. 7. PDPs depict the dynamic changes in predicted survival probability—defined as the likelihood of patients surviving for a specified duration—across the entire study cohort relative to follow-up time. This analytical approach is characterized by adjusting a single determinant while fixing all other clinical variables at their respective values in the training dataset, thereby isolating the independent effect of the target variable on survival outcomes.​

In Fig. 6, the y-axis denotes the predicted survival probability (ranging from 0 to 1), which reflects the model’s estimated probability that a patient will survive beyond a given time point (x-axis, expressed in months). Narrow and nearly overlapping labeled bands (representing confidence intervals) indicate that the overall predicted survival probability remains relatively consistent regardless of variations in the values of the corresponding variables. Conversely, wider and non-overlapping bands suggest that even minor modifications in variable values can lead to considerable fluctuations in predicted survival probability.​

Notably, several key clinical variables were identified to exert a significant impact on predicted survival probability, including AJCC stage, age, chemotherapy administration, tumour stage, M stage, marital status, liver metastasis, and radiation therapy. Specifically, patients with AJCC stage IV disease or those aged ≥ 80 years exhibited a more rapid decline in predicted survival probability compared to their counterparts (i.e., AJCC stage < IV or age < 80 years). A similar trend of accelerated survival probability reduction was observed in patients who did not receive chemotherapy, those with distant metastasis, those classified as M stage 1, unmarried patients, those with liver metastasis, and those who underwent radiation therapy.

#### Aggregated SurvSHAP values summary

SurvSHAP(t) represents an extension of the SHAP (SHapley Additive exPlanations) framework specifically customized for survival analysis scenarios, with its core function being to quantify the time-dependent contribution of each clinical feature to model predictive outcomes across different follow-up time points [12]. Grounded in the axiomatic basis of Shapley values derived from game theory, SurvSHAP(t) assigns a time-stratified importance score to each feature by assessing its marginal contribution to the prediction error when excluded from all potential feature subsets [13]. For survival analysis models such as the boosted tree (BT) model employed in this study, this process involves decomposing the predicted cumulative hazard function into individual feature-specific contributions. Consequently, the sum of all SurvSHAP(t) values corresponding to a single patient approximates the discrepancy between their individual predicted risk and the average risk of the entire study cohort [14].

**Fig. 8:**
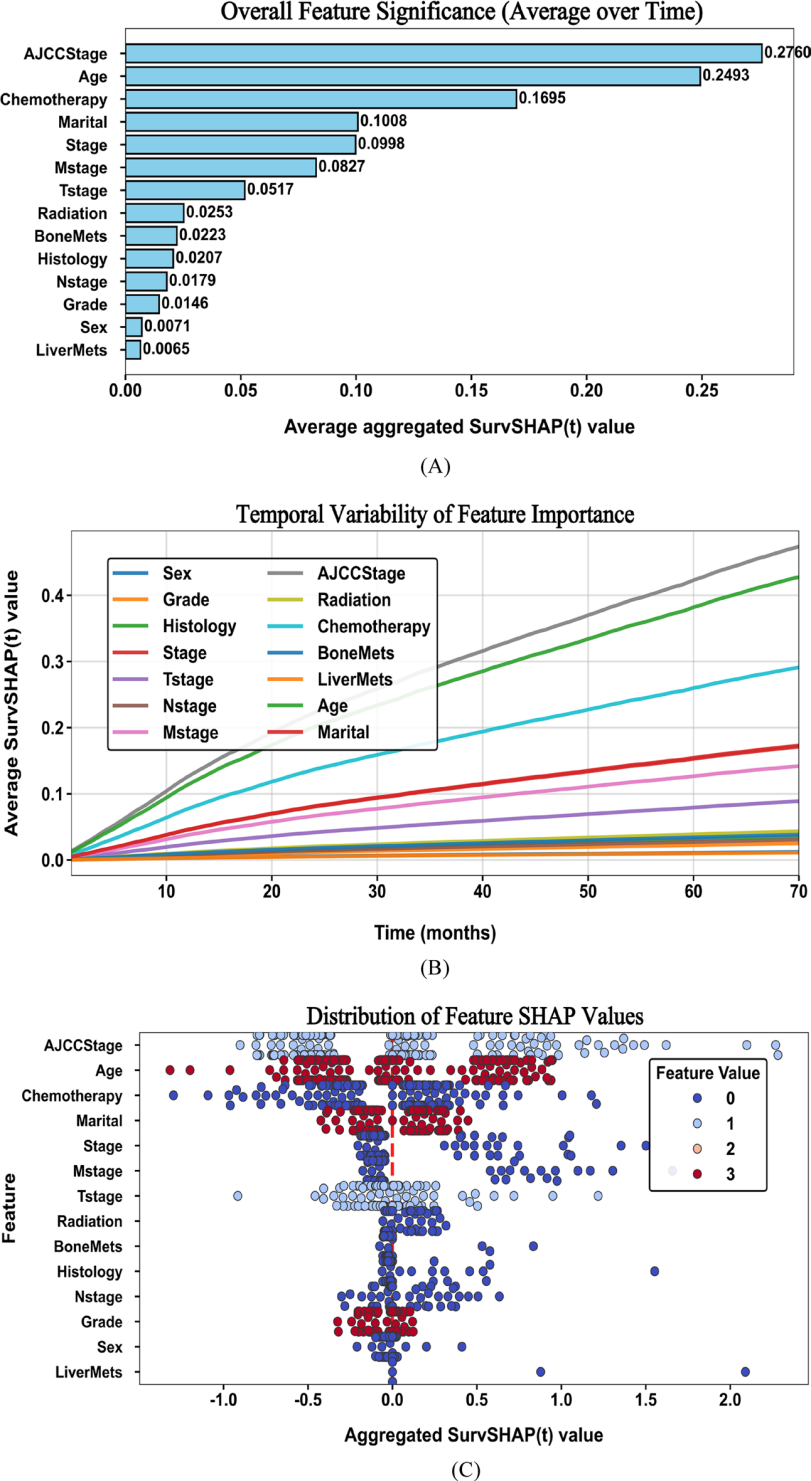

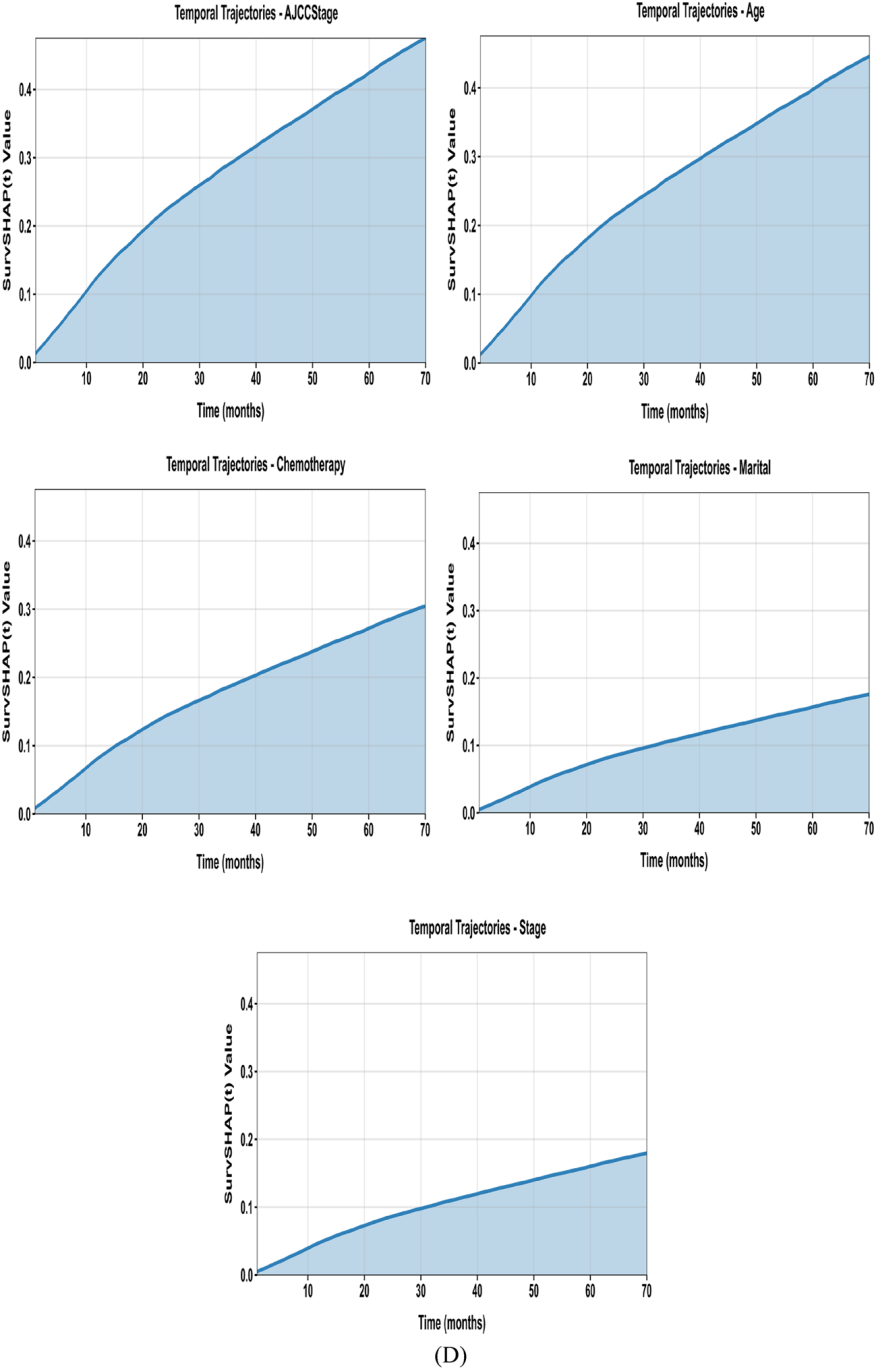

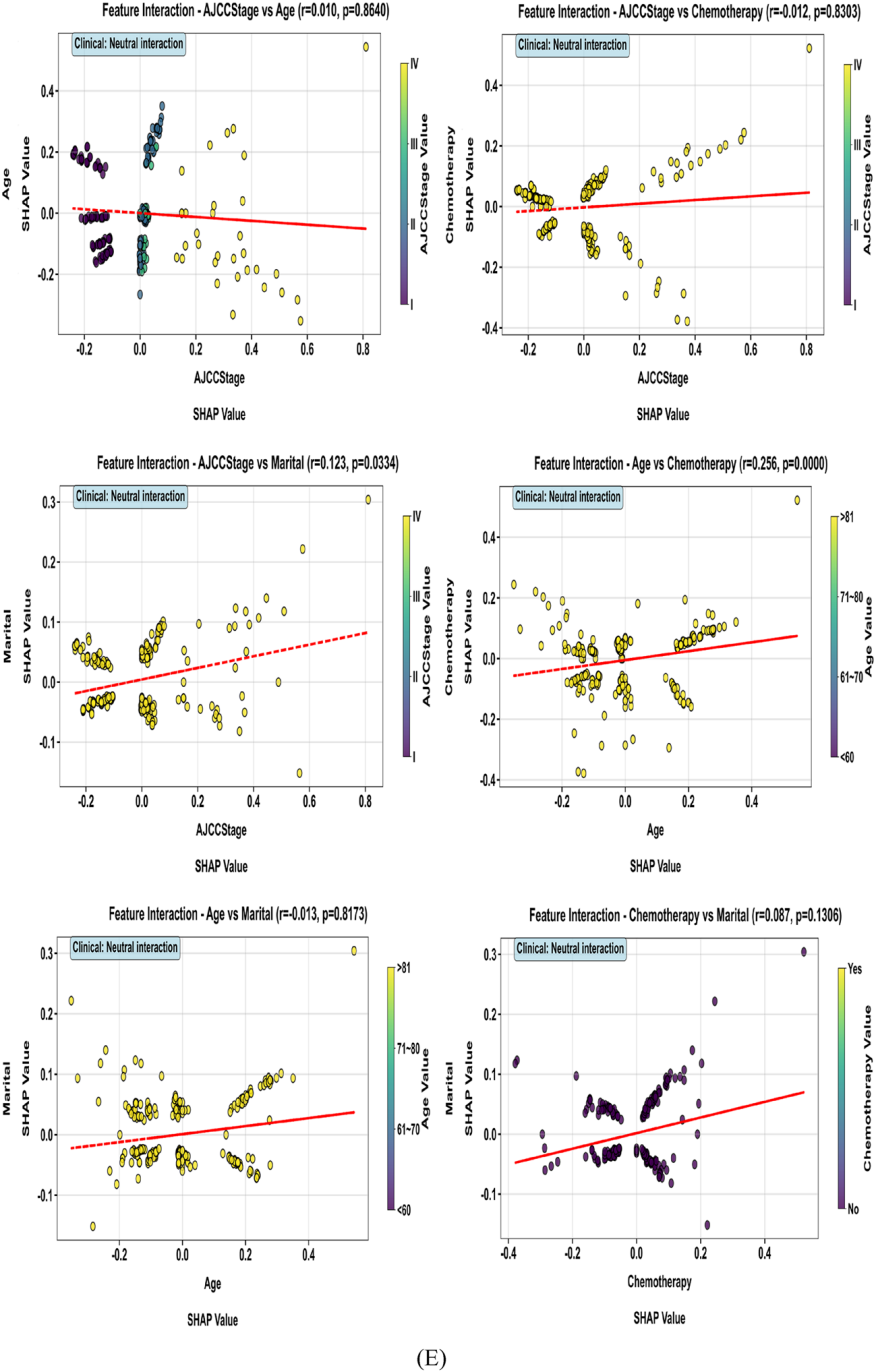
The SurvSHAP summary plot provides an overall interpretation of the global impact. **A** The length of the bar chart represents the overall significance of the variables. **B** The curve graph displays the cumulative importance of each variable. **C** Each point on the bee swarm plot represents a specific feature of a particular patient. The y-coordinate of the point is determined by the feature it represents, while the x-coordinate is determined by its impact on the model output. The features on the y-axis are sorted according to their significance. **D** Temporal trajectory curves of the top 5 influential features. **E** Two-way interaction plots of features

SurvSHAP summary plots for the BT model (encompassing 14 clinical features) were computed and visualized, with features ranked according to their overall impact on overall survival (OS). Figure 8A presents the global importance of each variable, which is defined as the mean absolute SurvSHAP(t) value across all follow-up time points and study observations. Figure 8B illustrates the temporal variability in the significance of each variable: the y-axis denotes the average absolute SurvSHAP(t) value at each individual time point, thereby highlighting the dynamic fluctuations in feature importance throughout the follow-up period.

In the bee swarm plot (Fig. 8C), variables are ordered in descending order based on their mean absolute SurvSHAP(t) values to reflect their overall importance. Each data point in the plot corresponds to a single patient’s SurvSHAP(t) value for a specific variable: the x-axis indicates both the magnitude and direction of the feature’s impact on OS prediction (positive values correspond to an elevated mortality risk, while negative values are indicative of a reduced risk). The color gradient (adopting a coolwarm palette) encodes the original feature values (red represents higher feature values, and blue denotes lower feature values) rather than the magnitude of the SurvSHAP(t) values. Among the 14 included features, AJCC stage, age, and chemotherapy administration exerted the most prominent overall impact on OS prediction, followed by marital status, tumour stage and M stage.

Figure 8D presents temporal trajectories of the top 5 influential features (AJCCStage, Age, Chemotherapy, Marital, Stage) over 0–70 months, stratified into early, mid-term, and late follow-up phases. AJCCStage and Age dominate across all follow-up periods. Chemotherapy exerts a consistent secondary influence throughout all phases, with stable protective effects. Marital status and Stage exhibit minimal prognostic impact in the early phase but gradually emerge as meaningful factors in mid-term and late follow-up, contributing to long-term survival outcomes.

Figure 8E includes two-way interaction plots features, with correlation coefficients (r) and statistical significance (p) analyzed to identify meaningful effect modifications. Among all feature pairs, two interactions reached statistical significance:

Age vs. Chemotherapy: A moderate positive correlation was observed (*r* = 0.256, *p* < 0.0001), indicating that the prognostic effect of chemotherapy is significantly modified by patient age—reflecting an age-dependent pattern of treatment benefit.

AJCCStage vs. Marital: A mild positive correlation was detected (*r* = 0.123, *p* = 0.0334), suggesting that high-risk marital status (Widowed) exerts a synergistic effect on the prognostic risk associated with advanced AJCCStage.

The remaining feature pairs showed only weak correlations with no statistical significance, indicating these features act independently on prognosis without meaningful interaction effects.

10-fold cross-validation and bootstrap resampling confirm stable feature ranking and significant interaction effects across cohorts, ensuring the reliability of the observed patterns.

## Discussion

This study constructed and validated seven overall survival (OS) prediction models for bladder cancer patients using a large cohort from the SEER database and an external validation cohort from Kashgar, Xinjiang, China. The boosting tree (bt) model ultimately demonstrated optimal performance in both predictive capability and interpretability. Through time-dependent feature importance analysis, partial dependency survival plots, and SurvSHAP analysis, systematically revealed key prognostic factors and their dynamic interaction patterns in bladder cancer patients, providing a tool for clinical personalized diagnosis and treatment that combines precision and transparency.

To further highlight the clinical added value of our model in real-world settings, we compared the optimal boosting tree (BT) model with three clinical nomograms for bladder cancer OS prediction: a nomogram integrating previous cancer history [15], a SEER/TCGA-based multi-database nomogram [16], and a high-grade bladder cancer post-surgical nomogram [17]. These nomograms were selected for their rigorous validation, and coverage of diverse clinical scenarios (general bladder cancer, molecular-integrated prediction, high-grade subtype)—ensuring a comprehensive and credible comparison.

The comparative analysis focused on three core dimensions: predictive performance, interpretability, and applicability. In terms of predictive performance, our BT model outperforms all three nomograms in the external validation cohort (Chinese Kashgar population). It exhibits better discriminative ability (reflected by higher C-index and AUC) and calibration (lower Brier score) than the selected nomograms, particularly in identifying high-risk patients who may benefit from intensified systemic therapy or closer follow-up.

In terms of interpretability, the BT model offers unique advantages over the three nomograms. All selected nomograms rely on Cox proportional hazards models or static feature weighting, providing fixed associations between variables and survival without insights into how prognostic factors change over time or contribute to individual patient outcomes. In contrast, our BT model integrates time-dependent feature importance, partial dependence survival plots, and SurvSHAP analysis to reveal dynamic patterns: AJCC stage is the dominant predictor for follow-up periods < 45 months, while age becomes the key prognostic factor after 45 months. This temporal perspective enables clinicians to develop personalized, staged management strategies—for example, prioritizing close monitoring of metastatic lesions in short-term high-risk patients and optimizing age-related comorbidity care in long-term survivors—an advantage unavailable in conventional static nomograms.

Regarding applicability, the BT model exhibits broader utility and cross-population robustness. It maintains stable performance across the Western (SEER) and Eastern (Chinese) populations, while the nomograms developed based on predominantly Western cohorts show notable decreases in predictive performance when applied to Chinese patients—likely due to epidemiological and treatment pattern differences. Additionally, one of the selected nomograms is limited to high-grade post-surgical patients, whereas our model covers diverse bladder cancer subtypes (TCP, SCN, EN, ADC) and treatment modalities (surgery, chemotherapy, radiation), enhancing its real-world applicability across different clinical settings.

Collectively, the comparative analysis with the three nomograms demonstrates that our BT model not only achieves superior predictive performance but also provides dynamic, clinically actionable interpretability and broad cross-population applicability—addressing the limitations of existing prognostic tools. These advantages enhance the model’s translation potential in real-world clinical practice, offering a more reliable tool for risk stratification and personalized treatment planning in bladder cancer patients.

### Analysis of model performance and clinical applicability

The results of this study demonstrate that although significant differences in tumor grade and histological subtype were observed across the three cohorts, the boosting tree model still exhibited stable and superior predictive performance in the internal and external validation sets, which further verified the strong generalization ability of the model and its insensitivity to minor baseline differences in clinical characteristics: AUC values for 1-year, 3-year, and 5-year OS all exceed 0.770, the comprehensive Brier score remains below 0.180, and the C-index consistently exceeds 0.72. This performance significantly outperformed traditional Cox proportional hazards models and other machine learning models (e.g., random survival forests, DeepSurv), confirming the BT model’s advantage in handling nonlinear associations and interaction effects within clinical data. By progressively optimizing residuals through gradient boosting, it more accurately captures complex relationships among variables like age, AJCC staging, and metastasis status, circumventing traditional models’ reliance on the “linear variable correlation” assumption [18, 19]. More importantly, the model’s performance showed no significant decline in the external validation cohort (Kashgar population in China), indicating strong generalization across Eastern and Western populations. This contrasts sharply with most bladder cancer prediction models built solely on data from a single region. Given the significant geographical epidemiological differences in bladder cancer (e.g., variations in smoking exposure and pathological subtype distribution between Asian and Western populations) [20], the multicenter validation design of this study effectively enhances the model’s clinical applicability, providing a reliable prognostic assessment tool for healthcare institutions across different regions.

Based on the variable screening results, the 14 independent prognostic factors identified in this study through LASSO regression and Cox multivariate analysis—including age, AJCC stage, M stage, bone metastasis, liver metastasis, chemotherapy, and radiotherapy—are highly consistent with existing clinical knowledge. For instance, AJCC staging, as the “gold standard” for bladder cancer staging systems, has been confirmed in multiple studies to possess strong prognostic predictive value [21]. Furthermore, distant metastasis status, such as bone and liver metastases, represents a significant indicator of reduced survival in advanced bladder cancer patients [22]. Notably, marital status emerged as an independent prognostic factor, potentially reflecting advantages in treatment adherence and social support among married patients. Previous research indicates that robust social support can indirectly improve cancer patient outcomes by reducing psychological stress and enhancing treatment compliance [23]. Incorporating these variables not only ensures the model’s clinical validity but also lays the groundwork for subsequent interpretability analysis.

### Clinical value of interpretability analysis

The core innovation of this study lies in overcoming the “black box” limitations of traditional machine learning models. By employing three complementary interpretability tools, the predictive logic of the BT model is transformed into intuitive clinical information:

Time-dependent feature importance reveals the dynamic patterns of prognostic factors: Among patients with survival < 45 months, AJCC staging is the most critical predictor, with the most significant decline in model performance after variable replacement (increased Brier score and largest decrease in AUC). Conversely, after 45 months of survival, age becomes the dominant influence. This finding provides crucial evidence for clinical stratified management: For patients with high short-term survival risk (e.g., AJCC Stage 4), intervention strategies should prioritize disease progression management (e.g., intensified systemic therapy, close monitoring of metastatic lesions). For long-term survivors, greater emphasis should be placed on managing age-related comorbidities (e.g., cardiovascular disease, renal function preservation) and implementing personalized follow-up protocols (e.g., adjusting examination frequencies for elderly patients to balance therapeutic benefits against healthcare burdens) [21]. This “time-stratified” prognostic assessment approach addresses the limitations of traditional “static prediction” models and better aligns with the dynamic nature of disease progression in clinical practice.

Partial dependency survival plots visually demonstrate how individual variables influence survival probability: for instance, patients with AJCC Stage 4 experience a significantly steeper decline in predicted survival probability compared to earlier stages, with similar trends observed in patients aged ≥ 80 years, those without chemotherapy, and those with liver metastases. These visualizations not only validate clinical observations—such as poorer outcomes in advanced-stage patients and chemotherapy’s survival-enhancing effects—but also enable physicians to rapidly assess individual patient risk levels. For instance, patients concurrently diagnosed with “AJCC Stage 4 + liver metastasis” can have their short-term mortality risk intuitively estimated via survival curves, allowing timely adjustment of treatment goals (e.g., shifting from curative to palliative care to improve quality of life).

SurvSHAP analysis quantifies feature contributions at both individual and population levels: The global importance ranking reveals that AJCC staging, age, and chemotherapy exhibit the highest mean absolute SurvSHAP values, making them core prognostic factors. The honeycomb plot further clarifies the directional association between variable values and risk (e.g., higher AJCC staging and older age correlate with more positive SurvSHAP values, indicating increased mortality risk; while chemotherapy corresponds to negative SurvSHAP values, indicating reduced risk). This “quantitative+qualitative” interpretation approach enables clinicians to understand “which factors matter” while clarifying “how they impact outcomes”. For example, for a 65-year-old patient with AJCC Stage 3 disease who did not receive chemotherapy, SurvSHAP values for each variable precisely pinpoint “no chemotherapy” as the primary risk contributor. This allows clinicians to prioritize considering an adjuvant chemotherapy regimen.

The deepened SurvSHAP analysis (Fig. 8D–E) uncovers non-trivial, clinically actionable insights beyond conventional oncology wisdom, delivering incremental scientific value to bladder cancer prognostic research. These findings not only refine our understanding of disease prognostic dynamics but also provide clear guidance for personalized clinical management.

The temporal trajectory patterns in Fig. 8D offer a staged prognostic framework that aligns with real-world clinical practice. AJCCStage and Age, as the dominant prognostic factors across all follow-up phases, highlight the need for phase-specific management priorities: in the early phase, AJCCStage-driven interventions (e.g., radical treatment for localized disease, systemic therapy for advanced stages) should take precedence, while mid-to-late follow-up should shift focus to age-related comorbidity management and long-term toxicity monitoring—reflecting Age’s sustained and increasing prognostic impact. Chemotherapy’s consistent secondary protective effect across all phases confirms its utility as a core therapeutic intervention regardless of follow-up stage, supporting the rationale for early initiation and maintenance of systemic therapy in eligible patients. Notably, the delayed prognostic influence of Marital status and Stage—emerging as meaningful factors only in mid-to-late follow-up—underscores the importance of dynamic risk reassessment. For long-term survivors, integrating social support status (e.g., identifying widowed patients at higher risk) and disease progression monitoring into routine follow-up can address previously unrecognized prognostic drivers, improving long-term outcomes.

The two statistically significant feature interactions identified in Fig. 8E further enhance personalized treatment decision-making. The moderate positive correlation between Age and Chemotherapy (*r* = 0.256, *p* < 0.0001) clarifies that chemotherapy’s benefit is not uniform across age groups. This finding supports age-adapted treatment strategies: middle-age patients (who typically exhibit better treatment tolerance) can safely receive standard chemotherapy to maximize curative potential, while elderly patients may benefit from de-escalated regimens or enhanced supportive care to balance efficacy and adverse effects. The mild positive correlation between AJCCStage and Marital status (*r* = 0.123, *p* = 0.0334) reveals a synergistic risk effect—advanced-stage patients with high-risk marital status (Widowed) face compounded prognostic challenges. For this vulnerable subgroup, integrating targeted supportive care (e.g., care coordination services, psychosocial support programs, and family engagement initiatives) alongside anti-tumor therapy can mitigate the adverse impact of social isolation, addressing a modifiable factor often overlooked in oncology management.

The robustness of these findings—validated via 10-fold cross-validation and bootstrap resampling—enhances clinician trust in translating these insights into routine care. Collectively, the deepened SurvSHAP analysis transforms descriptive prognostic observations into actionable, phase-specific, and subgroup-tailored treatment strategies, addressing unmet needs in bladder cancer management and highlighting the value of interpretable machine learning in uncovering hidden clinical patterns.

### Study limitations

Despite achieving meaningful results, this study faces several limitations in data sources. First, while the SEER database provides large-sample clinical data, key information (such as tumor molecular markers, specific chemotherapy regimen dosages, and details of comorbidities) is missing, potentially affecting the model’s predictive accuracy. Second, although the external validation cohort from Kashgar is valuable for testing the model’s cross-population generalization ability (especially for the Northwest Chinese ethnic population), its sample size (*n* = 713) is relatively small compared with the SEER training set (*n* = 15,760). This imbalance may reduce the statistical power to detect subtle differences in model performance and limit the representation of rare pathological subtypes and clinical characteristics to a certain extent. To mitigate these potential impacts, we strictly unified the inclusion/exclusion criteria, variable preprocessing strategies and prognostic factor screening logic between the two cohorts, supplemented the latest follow-up data to expand the Kashgar cohort from 650 to 713 cases, and used a comprehensive set of evaluation metrics (C-index, time-dependent AUC, integrated Brier score, KS test) to assess model performance—all metrics confirmed stable and robust predictive ability of the model in the external validation cohort. Third, the external validation cohort was derived solely from a single hospital in Kashgar, Xinjiang, China, featuring limited geographical representativeness. Future studies should incorporate cohorts from additional regions and multiple centers to further validate the model’s generalizability; we will also expand the sample size of the external validation cohort through multicenter collaboration to conduct large-sample validation and further improve the clinical applicability of the model in the Chinese population.

### Future outlook

Based on the findings of this study, future research may explore the following directions:

Refining model variables: Incorporate additional potential prognostic factors such as tumor molecular markers (e.g., FGFR3, NTRK fusions), immunotherapy-related indicators (e.g., PD-L1 expression), and lifestyle factors (e.g., smoking history, dietary patterns) to further enhance the model’s predictive accuracy [22, 23]; Expanding predictive dimensions: Current models predict overall survival only; future work should develop multi-outcome prediction models (e.g., concurrently predicting progression-free survival and treatment-related adverse events) to provide more comprehensive decision support for clinicians; Advancing clinical translation: Conducting prospective, multicenter studies to validate the model’s real-world efficacy; concurrently developing visualization tools for clinicians to lower the model’s adoption barrier, facilitating its transition from a “research tool” to a “clinical standard practice”.

In summary, the interpretable BT model constructed in this study demonstrates excellent performance and strong generalization capabilities in predicting OS for bladder cancer patients. Its interpretability analysis provides clear evidence for clinical stratification management and personalized treatment. Despite certain limitations, this model offers new research perspectives in the field of bladder cancer prognosis prediction and holds promise for improving patient outcomes and optimizing healthcare resource allocation.

## Conclusion

The interpretable boosting trees model developed in this study achieves both high accuracy and clinical interpretability in predicting OS for bladder cancer. Its validation results in the Chinese population provide a basis for cross-ethnic application. The model not only accurately predicts 1- to 5-year survival risks but also elucidates key influencing factors and dynamic patterns through visualization tools, offering reliable guidance for clinicians in developing personalized treatment plans (e.g., timing of surgery, metastasis monitoring strategies). This research validates the application value of interpretable machine learning in tumor prognosis, offering new tools and insights for precision diagnosis and treatment of bladder cancer.

## Supplementary Information

Below is the link to the electronic supplementary material.

Supplementary Material 1.

## Data Availability

The datasets used and analyzed during the current study are available from Surveillance, Epidemiology, and End Results (SEER) database of the National Cancer Institute (https://seerdataaccess.cancer.gov/seer-data-access). The data cannot be directly accessed on the SEER database’s official website. To access the data, one needs to download the SEER Stat software (https://seer.cancer.gov/seerstat/download/) from the official website. After registering and applying for an account and password (https://seerdataaccess.cancer.gov/seer-data-access), the data can be accessed by logging into the SEER Stat software using the obtained account and password. This complies with the journal’s data - sharing expectations.

## References

[1] Richters A, Aben KKH, Kiemeney LALM. The global burden of urinary bladder cancer: an update. World J Urol. 2020;38(8):1895–904. 31676912 10.1007/s00345-019-02984-4 PMC7363726

[2] Bray F, Ferlay J, Laversanne M, et al. Cancer Incidence in Five Continents: Inclusion criteria, highlights from X and the global status of cancer registration. Int J Cancer. 2015;137(9):2060–71. 26135522 10.1002/ijc.29670

[3] Cumberbatch MGK, Noon AP. Epidemiology, aetiology and screening of bladder cancer. Transl Androl Urol. 2019;8(1):5–11. 30976562 10.21037/tau.2018.09.11 PMC6414346

[4] Shangguan W, Hu J, Xie Y, et al. Conditional survival of trimodal therapy for nonmetastatic muscle-invasive bladder cancer: A SEER database analysis. Cancer Med. 2022;11(12):2356–65. 35301806 10.1002/cam4.4625 PMC9189453

[5] Siegel RL, Miller KD, Wagle NS, et al. Cancer statistics, 2023[J]. CA Cancer J Clin. 2023;73(1):17–48. 10.3322/caac.21763. 36633525 10.3322/caac.21763

[6] Yang S, Zhou H, Feng C, et al. Web-Based Nomograms for Overall Survival and Cancer-Specific Survival of Bladder Cancer Patients with Bone Metastasis: A Retrospective Cohort Study from SEER Database. J Clin Med. 2023;12(2):726. 36675655 10.3390/jcm12020726 PMC9865586

[7] Liu H, Zhang W, Zhang Y, et al. Mime: A flexible machine-learning framework to construct and visualize models for clinical characteristics prediction and feature selection. Comput Struct Biotechnol J. 2024;23:2798–810. 39055398 10.1016/j.csbj.2024.06.035 PMC11269309

[8] Guan C, Gong A, Zhao Y, et al. Interpretable machine learning model for new-onset atrial fibrillation prediction in critically ill patients: a multi-center study. Crit Care. 2024;28(1):349. 39473013 10.1186/s13054-024-05138-0 PMC11523862

[9] Martin SA, Townend FJ, Barkhof F, et al. Interpretable machine learning for dementia: A systematic review. Alzheimers Dement. 2023;19(5):2135–49. 36735865 10.1002/alz.12948 PMC10955773

[10] Ponce-Bobadilla AV, Schmitt V, Maier CS, et al. Practical guide to SHAP analysis: Explaining supervised machine learning model predictions in drug development. Clin Transl Sci. 2024;17(11):e70056. 39463176 10.1111/cts.70056 PMC11513550

[11] Cao R, Yang F, Ma SC, et al. Development and interpretation of a pathomics-based model for the prediction of microsatellite instability in Colorectal Cancer. Theranostics. 2020;10(24):11080–91. 33042271 10.7150/thno.49864 PMC7532670

[12] Dai H, Yu Z, Zhao Y, et al. Integrating machine learning models with multi-omics analysis to decipher the prognostic significance of mitotic catastrophe heterogeneity in bladder cancer. Biol Direct. 2025;20(1):56. 40259382 10.1186/s13062-025-00650-x PMC12012998

[13] Wang J, Kang Q, Tian S, et al. Development, Validation, and Deployment of a Time-Dependent Machine Learning Model for Predicting One-Year Mortality Risk in Critically Ill Patients with Heart Failure. Bioeng (Basel). 2025;12(5):511. 10.3390/bioengineering12050511 PMC12108603 40428130

[14] Sato K, Sazuka T, Arai T, et al. Machine learning analysis for detecting late recurrence and loss to follow-up after renal cell carcinoma surgery. BJUI Compass. 2024;5(10):950–6. 39416750 10.1002/bco2.425 PMC11479800

[15] Wang Z, Zhou Y, Guan C, et al. The impact of previous cancer on overall survival of bladder cancer patients and the establishment of nomogram for overall survival prediction. Med (Baltim). 2020;99(38):e22191. 10.1097/MD.0000000000022191 PMC7505356 32957347

[16] Zhang Y, Hong YK, Zhuang DW, et al. Bladder cancer survival nomogram: Development and validation of a prediction tool, using the SEER and TCGA databases. Med (Baltim). 2019;98(44):e17725. 10.1097/MD.0000000000017725 PMC6946294 31689813

[17] Li Y, Chen T, Fu B, et al. Survival nomogram for high-grade bladder cancer patients after surgery based on the SEER database and external validation cohort. Front Oncol. 2023;13:1164401. 37397381 10.3389/fonc.2023.1164401 PMC10313206

[18] Yagin FH, Alkhateeb A, Raza A, et al. An Explainable Artificial Intelligence Model Proposed for the Prediction of Myalgic Encephalomyelitis/Chronic Fatigue Syndrome and the Identification of Distinctive Metabolites. Diagnostics (Basel). 2023;13(23):3495. 38066735 10.3390/diagnostics13233495 PMC10706650

[19] Javanmard Z, Zarean Shahraki S, Safari K, et al. Artificial intelligence in breast cancer survival prediction: a comprehensive systematic review and meta-analysis. Front Oncol. 2025;14:1420328. 39839787 10.3389/fonc.2024.1420328 PMC11747035

[20] Zhang LY, Wang P, Wang YB, et al. Global, regional, and national burden of bladder cancer, 1990–2019: an age-period-cohort analysis based on the Global Burden of Disease 2019 study. Public Health. 2024;236:193–203. 39265377 10.1016/j.puhe.2024.07.027

[21] Nouh MR, Ezz Eldin O. Precise vesical wall staging of bladder cancer in the era of precision medicine: has it been fulfilled? Abdom Radiol (NY). 2025;50(7):3084–91. 39725735 10.1007/s00261-024-04786-8

[22] Witjes JA, Bruins HM, Cathomas R, et al. European Association of Urology Guidelines on Muscle-invasive and Metastatic Bladder Cancer: Summary of the 2020 Guidelines. Eur Urol. 2021;79(1):82–104. 32360052 10.1016/j.eururo.2020.03.055

[23] Krajc K, Miroševič Š, Sajovic J, et al. Marital status and survival in cancer patients: A systematic review and meta-analysis. Cancer Med. 2023;12(2):1685–708. 35789072 10.1002/cam4.5003 PMC9883406

