# Supplementary material for: Interpretable machine learning models for bladder cancer overall survival prediction development and external validation via SEER database and Chinese cohort analysis
Source: Discov Oncol. 2026 May 20;17:1091. doi: 10.1007/s12672-026-05099-6 (PMC13407816; doi:10.1007/s12672-026-05099-6)
Supplement: Supplementary file 1 — Supplementary Material 1. [file 12672_2026_5099_MOESM1_ESM.docx]

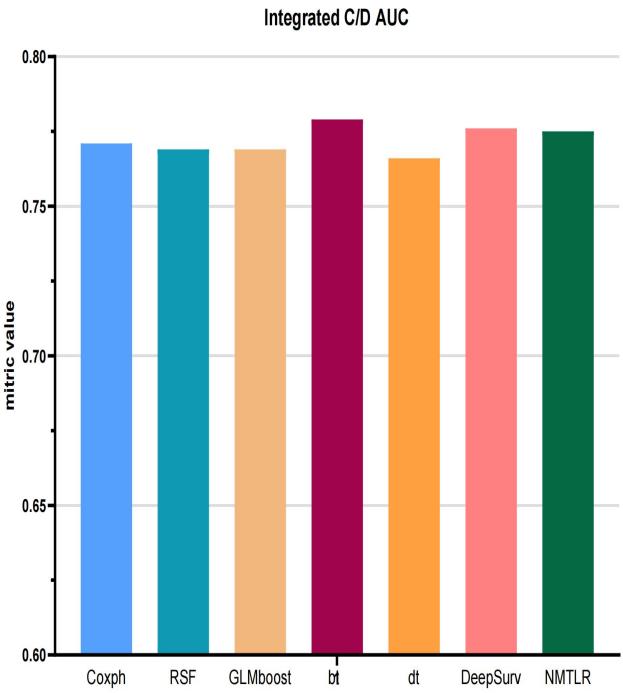

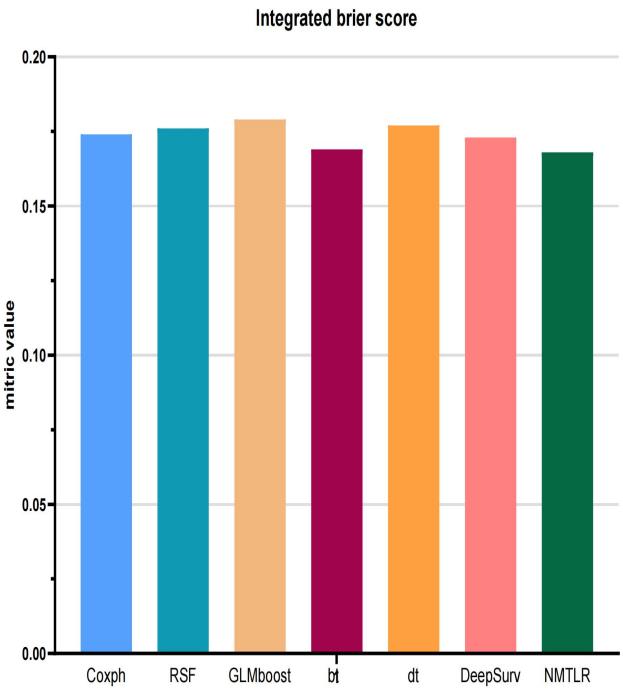


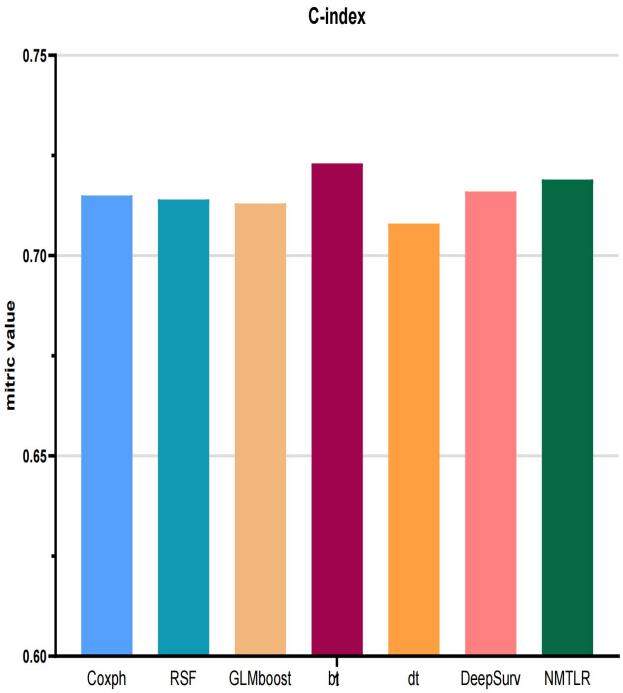

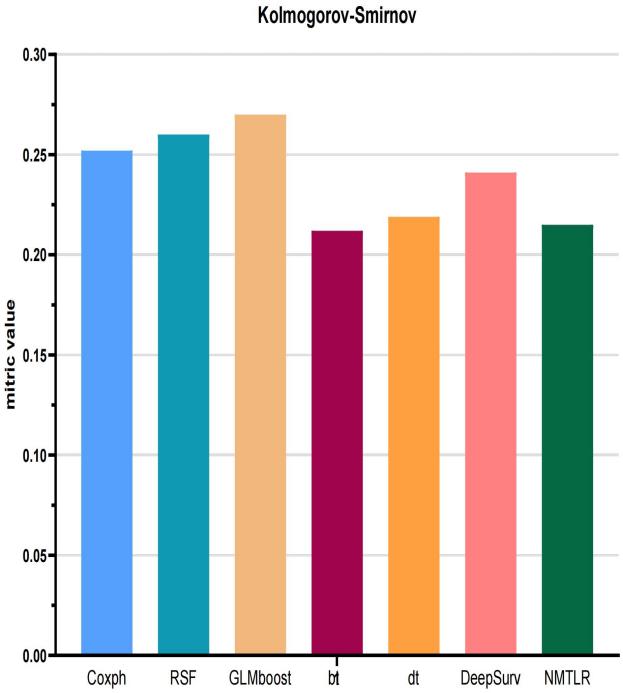


(A)


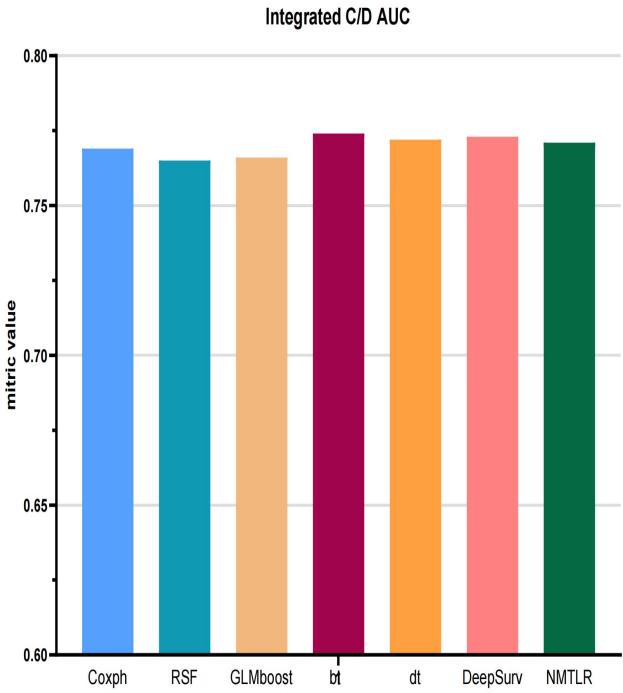

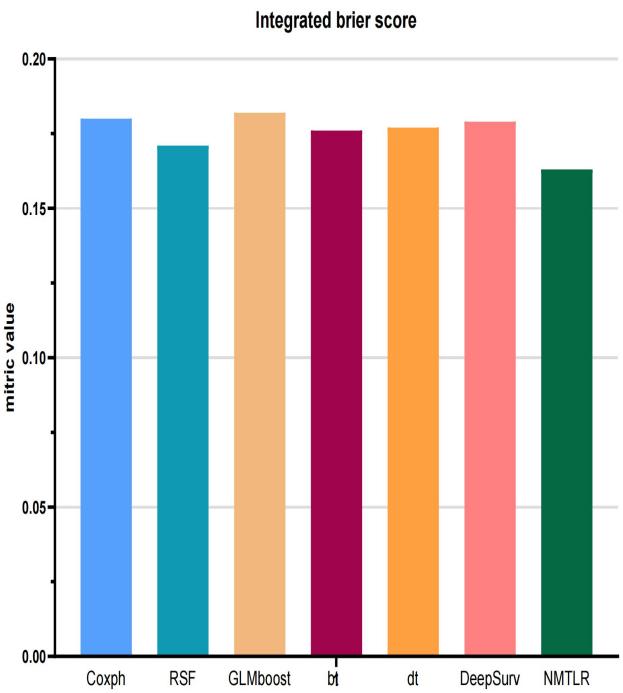


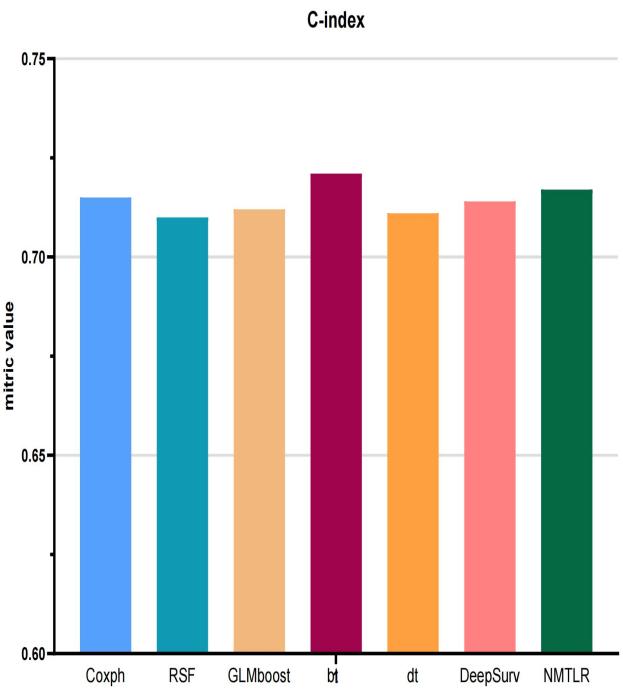

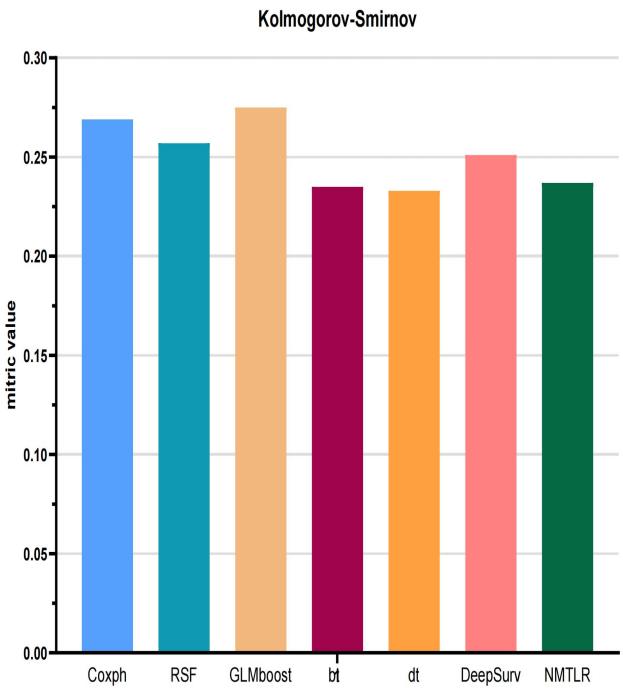


(B)

Figure S1 Model performance was displayed in the form of bar plots (A)Validation set; (B)External validation set


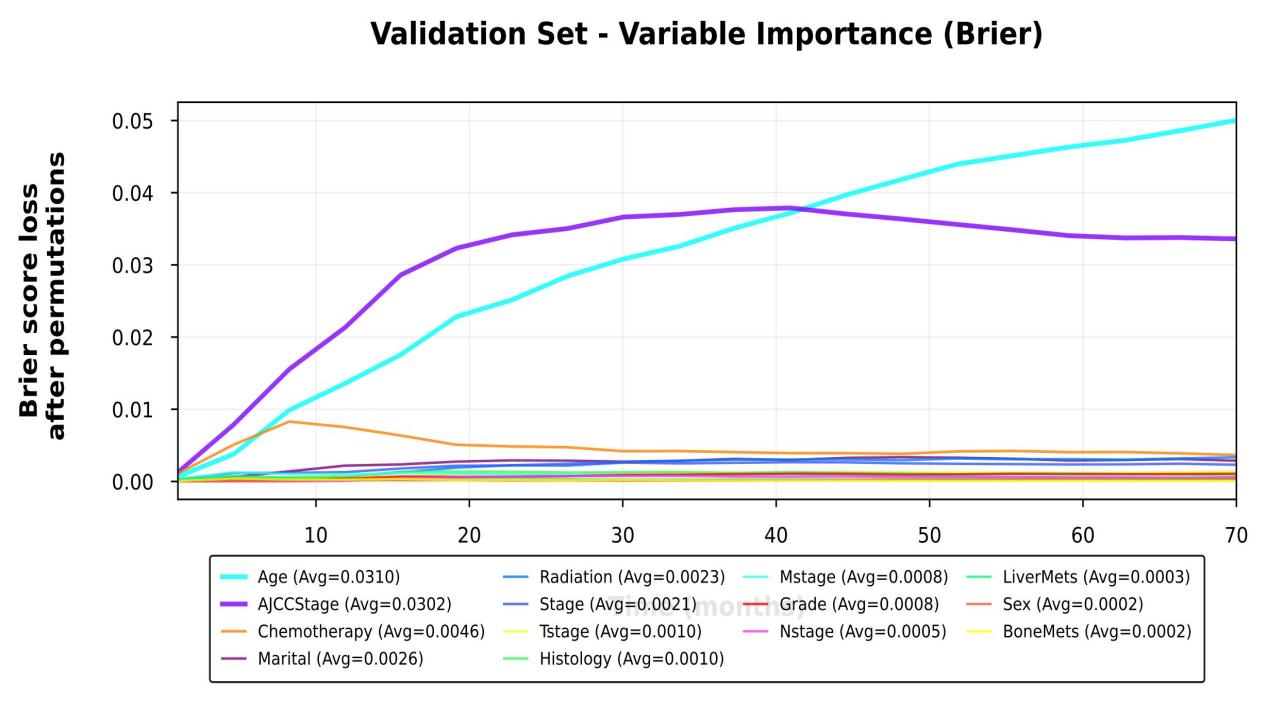


(A)


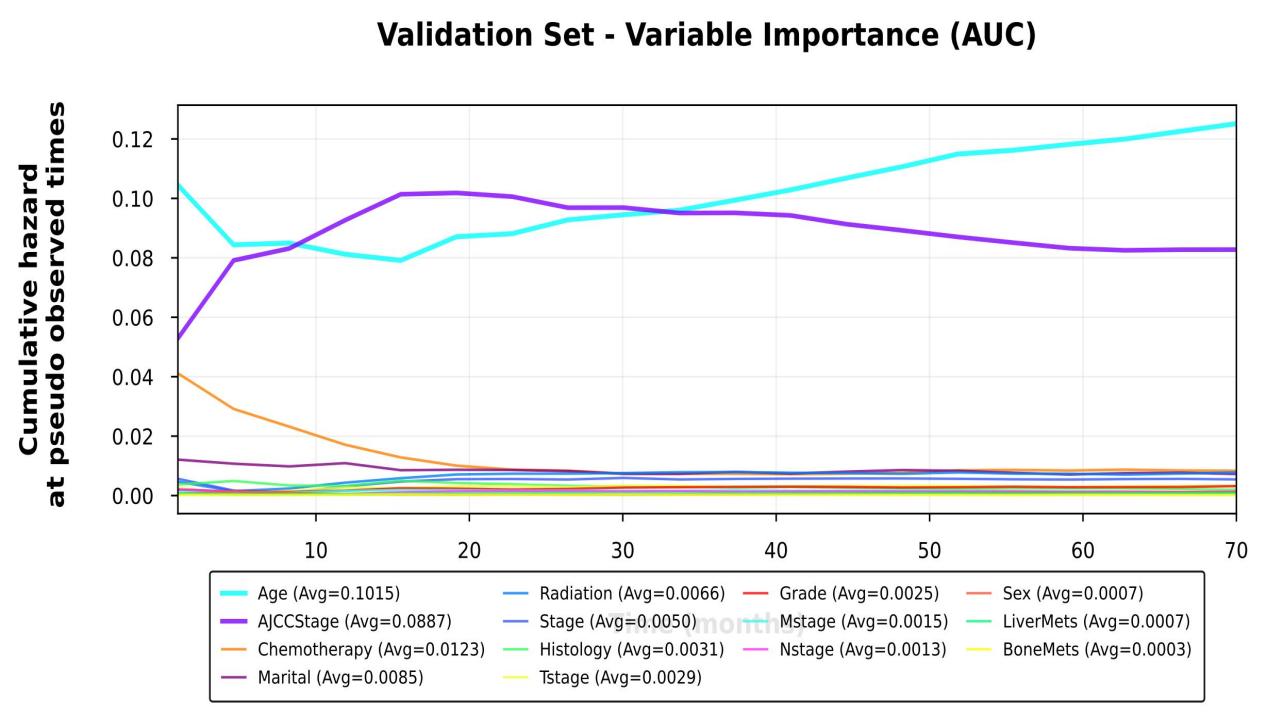


(B)


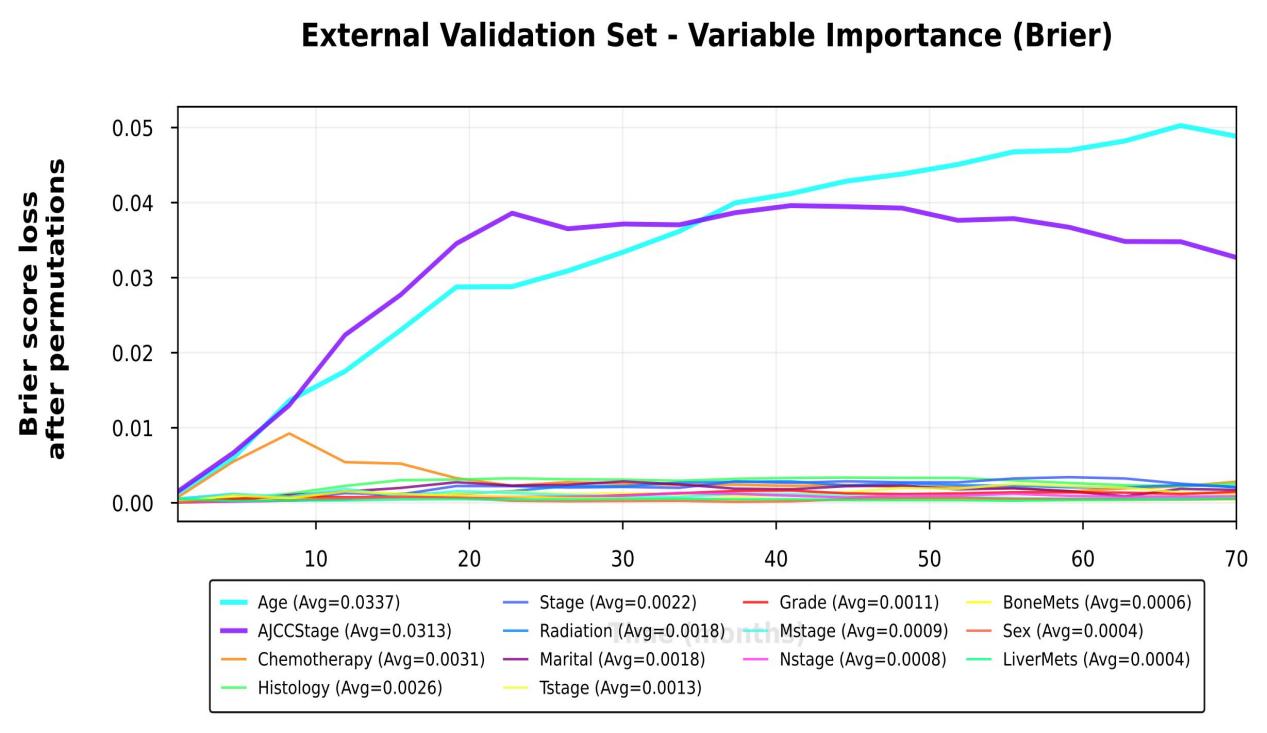


(C)


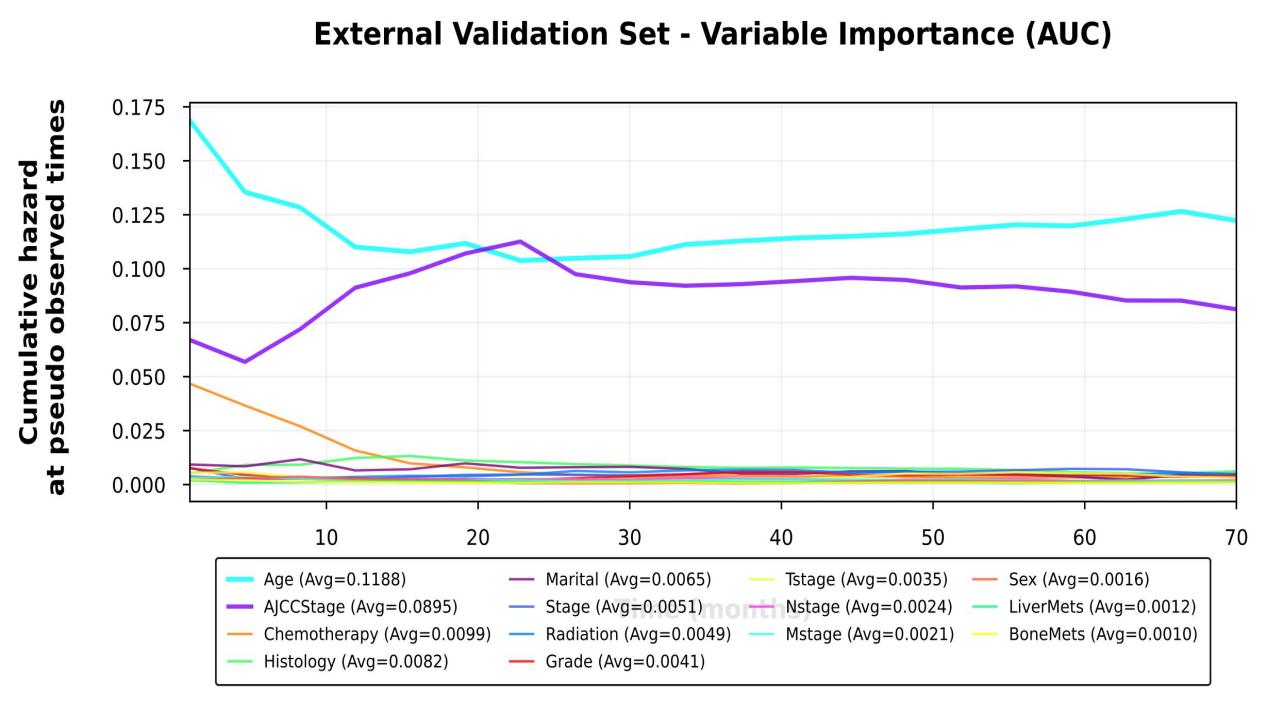


(D)

Figure S2 Time-­dependent feature importance, (A) The Brier score loss after permutation for the validation set; (B) the C/D AUC loss after permutation for the validation set; (C) The Brier score loss after permutation for the external validation set; (D) the C/D AUC loss after permutation for the external validation set
